# Comparative Effectiveness of Focused Versus Radial Extracorporeal Shockwave Therapy and Leukocyte-Poor Versus Leukocyte-Rich Platelet-Rich Plasma for Lateral Epicondylitis: A Network Meta-Analysis

**DOI:** 10.3390/jcm15176679

**Published:** 2026-08-28

**Authors:** Jun Min Cha, Seo Yeon Yoon, Yong Wook Kim, Jong Mi Park, Sang Chul Lee

**Affiliations:** Department and Research Institute of Rehabilitation Medicine, Yonsei University College of Medicine, 50-1 Yonsei-ro, Seodaemun-gu, Seoul 03722, Republic of Korea; ratedrko@yuhs.ac (J.M.C.); syyoon23@yuhs.ac (S.Y.Y.); ywkim1@yuhs.ac (Y.W.K.)

**Keywords:** tennis elbow, extracorporeal shockwave therapy, platelet-rich plasma, network meta-analysis, rehabilitation

## Abstract

**Background/Objectives:** Lateral elbow tendinopathy (LET) causes chronic pain and functional limitation, and the evidence base is confounded by the “lumping” of biologically and physically distinct interventions. We conducted a systematic review and network meta-analysis (NMA) distinguishing focused (fESWT) from radial (rESWT) extracorporeal shockwave therapy (ESWT) and leukocyte-rich (LrPRP) from leukocyte-poor (LpPRP) platelet-rich plasma (PRP) to establish a precise hierarchy of treatment efficacy. **Methods:** We searched PubMed, EMBASE, Cochrane, Scopus, and Web of Science from inception to 8 December 2025, with the search updated on 12 August 2026. Twenty-three randomized controlled trials (1484 participants) evaluating fESWT, rESWT, LrPRP and LpPRP were included. Standardized mean differences (SMDs) were calculated using a random-effects model, and treatments were ranked by the surface under the cumulative ranking curve (SUCRA). **Results:** fESWT ranked highest for grip strength overall (SUCRA: 82.6) and in the short term (≤3 months; 78.4). For upper-limb disability (Disabilities of the Arm, Shoulder and Hand), rESWT ranked highest overall (92.9) and in the long term (≥6 months; 94.0), while fESWT ranked lowest (19.9). rESWT was also most effective for pain at rest (Visual Analogue Scale; 86.6) and fESWT for disease-specific function (Patient-Rated Tennis Elbow Evaluation; 81.9), both significantly superior to placebo. The LrPRP advantage did not persist when trials at high risk of bias were excluded. **Conclusions:** Clinical outcomes in LET are influenced by the specific subtypes of ESWT and PRP. rESWT was most effective for pain at rest and long-term upper-limb function, whereas fESWT was superior for short-term grip strength and disease-specific function. The apparent benefit of LrPRP was less robust and should be regarded as preliminary, and treatment selection should be tailored to a patient’s recovery priorities.

## 1. Introduction

Lateral epicondylitis—often referred to as tennis elbow and increasingly described as lateral elbow tendinopathy (LET)—is a common musculoskeletal disorder characterized by pain and tenderness over the lateral epicondyle, most frequently involving the extensor carpi radialis brevis (ECRB) tendon and the common extensor origin [1]. It affects approximately 1% to 3% of adults, with peak occurrence typically reported in middle-aged individuals [2]. Although historically considered an inflammatory condition, LET is now widely regarded as a degenerative overuse tendinopathy associated with repetitive mechanical loading and a failed reparative response, with characteristic angiofibroblastic hyperplasia and collagen disorganization changes rather than classic inflammatory cell infiltration [3].

While tennis-related activities are a well-known trigger, LET is also highly relevant in occupational contexts that require repetitive or forceful upper-limb tasks [4]. If not managed effectively, symptoms may become chronic, leading to significant functional limitations such as reduced pain-free grip strength and difficulty in daily activities [5]. Furthermore, persistent LET can contribute to secondary complications, including myofascial pain syndrome in the neck and shoulder regions, muscle weakness throughout the kinetic chain, and a decline in psychological well-being or quality of life [6]. Although the natural history can be favorable for some, refractory cases may ultimately necessitate invasive surgical intervention [7].

Therefore, implementing appropriate interventions at the right time is crucial to prevent these long-term complications. A broad range of treatment options is currently used, including exercise-based physiotherapy, non-steroidal anti-inflammatory drugs (NSAIDs), bracing, and injection therapies such as corticosteroids and platelet-rich plasma (PRP), as well as physical modalities such as extracorporeal shockwave therapy (ESWT) [5,8,9]. However, the relative effectiveness of these interventions remains debated due to significant heterogeneity in treatment protocols [10].

Most importantly, many existing evidence syntheses have historically “lumped” biologically or physically distinct interventions together. For instance, focused ESWT (fESWT) and radial ESWT (rESWT) differ fundamentally in energy distribution and tissue penetration, while leukocyte-rich (LrPRP) and leukocyte-poor (LpPRP) formulations exert different inflammatory and reparative signaling profiles [8,11]. Failing to distinguish these subtypes potentially obscures clinically meaningful differences in efficacy.

Accordingly, this study aims to comprehensively evaluate and compare the effectiveness of diverse interventions for LET using a network meta-analysis (NMA). By integrating both direct and indirect evidence, NMA enables a robust comparison and ranking of multiple treatment modalities. Through a granular classification of ESWT (fESWT vs. rESWT) and PRP (Lr vs. Lp), our analysis seeks to provide a more precise, clinically actionable hierarchy of treatment efficacy. Ultimately, these findings are expected to serve as a cornerstone for developing specific clinical guidelines and optimizing individualized treatment strategies for patients with tennis elbow.

## 2. Materials and Methods

### 2.1. Compliance with Guidelines

This study followed the Preferred Reporting Items for Systematic Reviews and Meta-Analyses (PRISMA) 2020 statement and its extension for network meta-analyses (PRISMA-NMA) for its conduct and reporting (Table S1) [12,13]. This review was registered in the International Prospective Register of Systematic Reviews (PROSPERO) (registration number: CRD420261294625). The registered outcomes were pain (Visual Analogue Scale, VAS), upper-limb disability (Disabilities of the Arm, Shoulder and Hand, DASH), disease-specific function (Patient-Rated Tennis Elbow Evaluation, PRTEE), and grip strength. The record was registered after the literature search had commenced; the registration is therefore retrospective. Institutional review board approval was not required for this study.

### 2.2. Search Strategy

A comprehensive systematic literature search was executed across several electronic databases, namely PubMed, EMBASE, Cochrane, Scopus, and Web of Science. The search encompassed all available records from their respective inception dates to 8 December 2025 and was re-run on 12 August 2026 to capture newly indexed reports; no additional eligible trial was identified (Table S2). Furthermore, we manually scrutinized the bibliographies of the selected trials and pertinent review papers to ensure no relevant studies were overlooked. For a full description of the search strings used, please refer to Table S2.

### 2.3. Inclusion and Exclusion Criteria

Eligibility for this review was determined according to the PICOS (Population, Intervention, Comparison, Outcomes, and Study Design) framework. The target population consisted of individuals with lateral epicondylitis (tennis elbow). We included studies evaluating the following therapeutic options: focused and radial extracorporeal shockwave therapy, and platelet-rich plasma (both leukocyte-poor and leukocyte-rich varieties). ESWT arms were classified as focused when a generator producing a convergent, deeply focused pressure field was used, and as radial when a ballistic device producing a divergent, superficially distributed pressure wave was used. PRP arms were classified as leukocyte-rich or leukocyte-poor according to the explicit designation of the original authors; where the terminology was not stated, classification was based on the reported preparation system and centrifugation protocol (Table S3). Two reviewers classified every PRP arm independently, with complete agreement (Cohen’s κ = 1.00). These interventions were compared against conservative exercise, wait-and-see approaches, or placebos (including saline injections and sham ESWT), as well as direct comparisons between active treatments. Because sham ESWT, saline injection, and conservative or wait-and-see care all represent non-active comparators without a specific biological or mechanical treatment effect, they were combined into a single placebo/control node. Consistent with the registered protocol, pain (VAS at rest), disease-specific function (PRTEE), upper-limb disability (DASH), and grip strength were analysed as outcomes of interest; adverse events were assessed narratively. Only randomized controlled trials (RCTs) were considered for inclusion. Two investigators independently screened all titles and abstracts against these criteria and then assessed the full texts of potentially eligible reports; disagreements at either stage were resolved by discussion with a third investigator.

### 2.4. Data Extraction and Quality Assessment

Information retrieval was performed independently by two researchers using a standardized data extraction template. Any inconsistencies in the extracted data were settled through consultation with a third investigator. The gathered data points encompassed the primary author, publication year, trial design, cohort size, and patient demographics (including average age, sex distribution, duration of symptoms, and initial functional ratings), alongside specifics of the interventions, controls, and all measured outcomes. In cases of ambiguous or missing information, we contacted the original authors for clarification or additional data. To evaluate the methodological quality, the Revised Cochrane Risk of Bias Tool for Randomized Trials (RoB2) was employed [14]. Furthermore, the Confidence in Network Meta-Analysis (CINeMA) framework was used to determine the certainty of the evidence for the key outcomes, implemented in the CINeMA web application (Institute of Social and Preventive Medicine, University of Bern, Bern, Switzerland; https://cinema.ispm.unibe.ch, accessed on 12 August 2026) [15].

### 2.5. Data Synthesis and Statistical Analysis

To handle studies with missing information, we calculated the mean and standard deviation (SD) for changes from baseline following the guidelines in Chapter 6 of the Cochrane Handbook (version 6.4) [16]. Outcomes were quantified as standardized mean differences (SMDs) accompanied by 95% confidence intervals (CIs) using a random-effects model. We performed pairwise meta-analyses for comparisons involving two or more studies, with I^2^ and τ^2^ used to quantify statistical heterogeneity; values of I^2^ ≥ 50% were considered to represent substantial heterogeneity, and heterogeneity statistics for every network are reported in Table S4. Provided that the transitivity assumption was not significantly violated, a random-effects NMA was conducted. The geometry of each network was examined using network plots in which each node represents a treatment, node size is proportional to the number of participants randomised to that treatment, and edge width is proportional to the number of trials providing direct evidence. Network consistency was verified by evaluating the differences between direct and indirect evidence through node-splitting methods and the design-by-treatment interaction model [17,18]. The surface under the cumulative ranking curve (SUCRA) was used to rank the comparative effectiveness of the treatments [19]. Subgroup analyses were performed according to follow-up duration (≤3 months and ≥6 months). A pre-specified sensitivity analysis was performed by repeating every network after excluding the trials judged to be at high risk of bias (Table S5). We assessed potential publication bias via Egger’s test and visual inspection of funnel plot symmetry. All networks were estimated in R (version 4.5.2; R Foundation for Statistical Computing, Vienna, Austria) with the netmeta package (version 3.6.1), using restricted maximum likelihood estimation of the between-study variance.

## 3. Results

### 3.1. Study Identification and Characteristics

**Figure 1:**
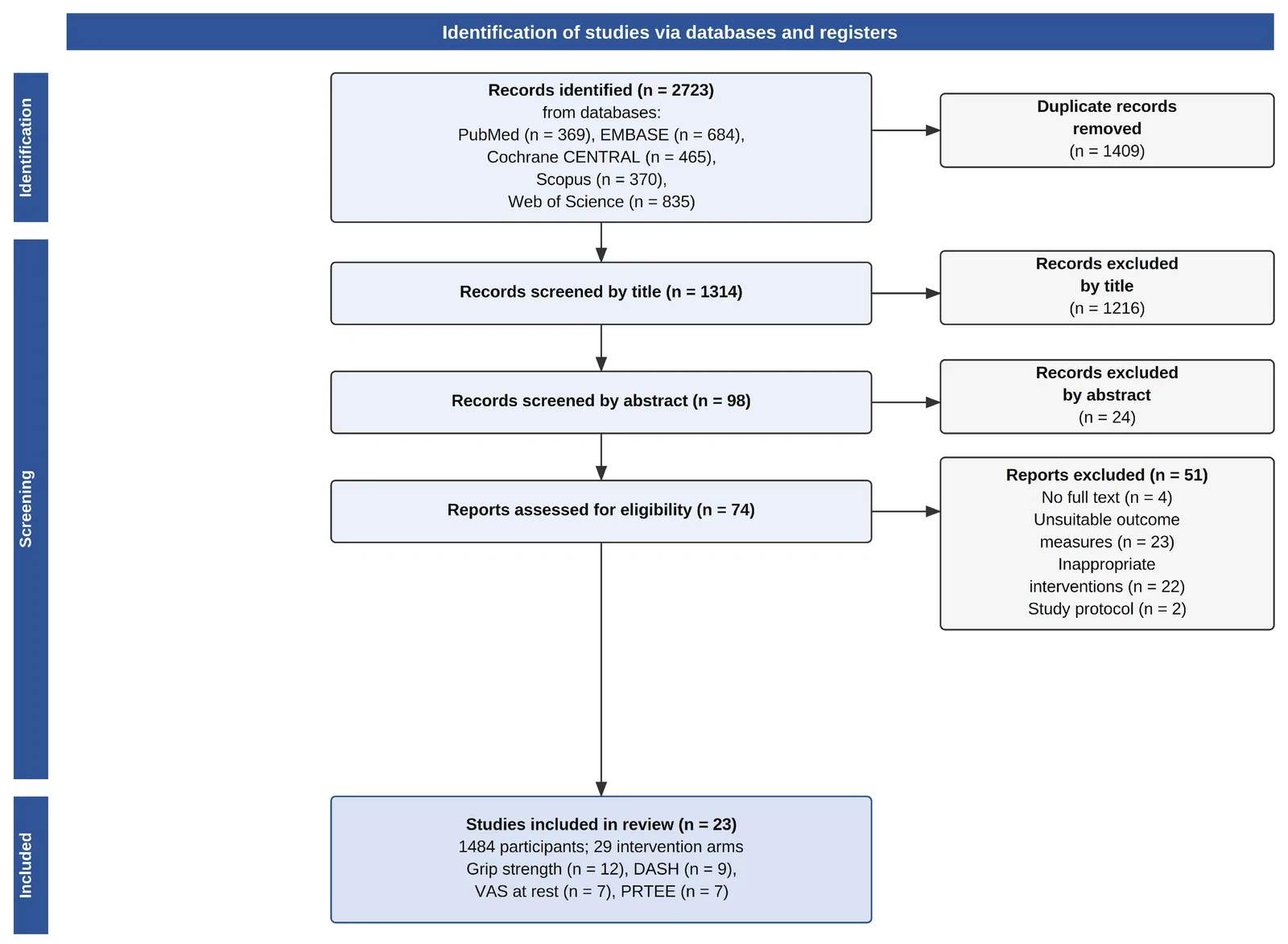
PRISMA (Preferred Reporting Items for Systematic Reviews and Meta-Analyses) flowchart illustrating the screening and selection process of the included studies.

A flowchart illustrating the study selection process is shown in Figure 1. Of the 2723 records identified, 1409 duplicates were removed. Of the 1314 records screened by title, 1216 were excluded; of the 98 records screened by abstract, a further 24 were excluded. Among the 74 reports assessed for eligibility, 51 were excluded for various reasons, including lack of full text (*n* = 4), unsuitable outcome measurements (*n* = 23), inappropriate interventions (*n* = 22), and study protocols (*n* = 2). Ultimately, 23 studies met the eligibility criteria and were included in the meta-analysis, comprising 1484 participants allocated to the treatments represented in the networks, with sample sizes ranging from 28 [20] to 231 [21] participants per study. The average age of the participants ranged from 41.4 [22] to 52.6 [23] years, and all studies included both male and female participants. The follow-up duration varied from one to 104 weeks, and the included studies spanned acute to chronic stages of tennis elbow. Because several trials directly compared two active interventions (e.g., fESWT vs. rESWT), the 23 included studies contributed a total of 29 eligible intervention arms: 11 arms of fESWT, 11 of rESWT, three of LrPRP, and four of LpPRP. Consequently, the sum of the arm counts exceeds the number of studies. Of the 23 included studies, 12 contributed data to the grip strength network, nine to the DASH network, seven to the VAS network and seven to the PRTEE network; several trials contributed to more than one network, and 1133 participants contributed data to at least one of the four analysed networks. Because Stania et al. reported two independent cohorts stratified by symptom duration, and because three-arm trials contribute to more than one pairwise comparison, the number of comparisons shown in each network plot exceeds the number of contributing trials. Three trials (Collins et al., Rompe et al. and Montalvan et al.) met the eligibility criteria but reported pain only during resisted wrist extension or on activity, or reported no dispersion measure, and therefore could not be entered into any of the four analysed networks; they are retained in the qualitative synthesis and in the risk-of-bias assessment. A summary of the characteristics of the included studies is presented in Table S6, and the classification of every PRP arm is given in Table S3.

### 3.2. Risk of Bias and Publication Bias Assessment

All 23 studies were randomized trials that provided details of their randomization methods. To minimize bias related to the intended intervention and measurement, 16 studies implemented a sham or placebo intervention in the control group. Specifically, 11 studies utilized sham devices to maintain high-quality blinding, while five studies employed saline as a placebo injection to ensure the validity of the comparative outcomes [20,22,23,24,25,26,27,28,29,30,31,32,33,34,35,36]. Two studies were identified as having a risk of missing outcome data bias, attributed to factors such as high attrition or early study termination [27,33]. Similarly, potential measurement bias was noted in four studies, frequently linked to the unblinded assessment of subjective outcomes like pain and functional scores [37,38,39,40]. Furthermore, most of the included studies explicitly referenced a pre-registered protocol or clinical trial registry entry, facilitating a low risk of bias in the selection of reported results by allowing a comparison between planned and observed outcomes. Overall, the included studies consisted of 17 RCTs at low risk of bias [20,21,22,23,24,25,26,28,29,30,31,32,34,35,36,41,42], three RCTs with some concerns [27,38,39], and three RCTs deemed to have a high risk of bias [33,37,40]. Figure S1 illustrates a traffic light diagram that summarizes the risk-of-bias assessments across the included studies. Funnel plots and Egger’s tests are presented for all eight networks with sufficient comparisons (Figure S2A–H). No evidence of small-study effects was found for grip strength (overall *p* = 0.635; ≤3 months *p* = 0.482), DASH (*p* = 0.614; *p* = 0.750) or PRTEE (*p* = 0.298; *p* = 0.354), whereas Egger’s test was significant for pain at rest (VAS overall *p* = 0.001; ≤3 months *p* = 0.003), indicating possible small-study effects for this outcome; the VAS results should therefore be interpreted with corresponding caution.

### 3.3. Evaluation of Inconsistency

Inconsistency was evaluated using both local (node-splitting) and global (design-by-treatment interaction) approaches. Although most loops were consistent, several individual comparisons showed local inconsistency, particularly within the grip strength and VAS networks (Table S7A). The global test identified significant inconsistency for grip strength (χ^2^ = 19.32, df = 2, *p* < 0.001) and for pain at rest (VAS; χ^2^ = 10.22, df = 1, *p* = 0.001), as well as for the short-term and long-term grip strength networks and the short-term VAS network (all *p* < 0.01) (Table S7B); results for these outcomes should therefore be interpreted with caution. No significant global inconsistency was detected for DASH or PRTEE at any follow-up window, and the long-term VAS and PRTEE networks contained no closed loop, so inconsistency could not be evaluated for them. Between-study heterogeneity was substantial for the DASH (I^2^ 91–94%) and PRTEE (I^2^ 80–91%) networks and for the overall and short-term VAS networks (I^2^ 78–86%), whereas it was negligible for grip strength overall and in the short term (I^2^ 0–7%); the full heterogeneity statistics are reported in Table S4.

### 3.4. Effects on Grip Strength

**Figure 2:**
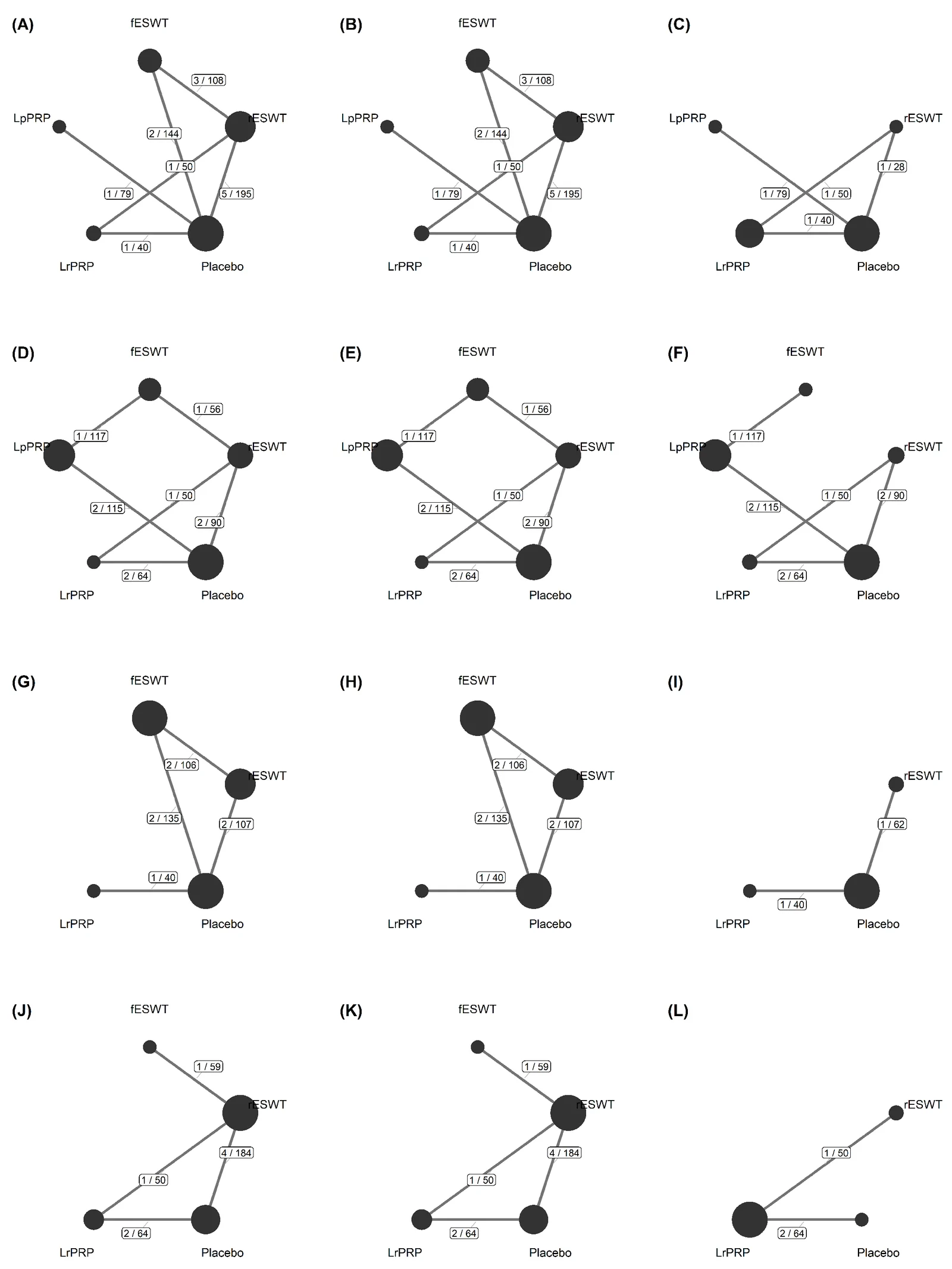
Network plots of treatments for tennis elbow. (**A**) Grip strength; (**B**) Grip strength (≤3 months); (**C**) Grip strength (≥6 months); (**D**) DASH; (**E**) DASH (≤3 months); (**F**) DASH (≥6 months); (**G**) VAS; (**H**) VAS (≤3 months); (**I**) VAS (≥6 months); (**J**) PRTEE; (**K**) PRTEE (≤3 months); (**L**) PRTEE (≥6 months). Node size is proportional to the total number of patients randomised to each treatment and edge width to the number of trials contributing to each direct comparison; the label adjacent to each edge shows the number of trials and the total number of patients for that comparison (trials/patients). DASH, Disabilities of the Arm, Shoulder and Hand; PRTEE, Patient-Rated Tennis Elbow Evaluation; VAS, Visual Analogue Scale.

**Figure 3:**
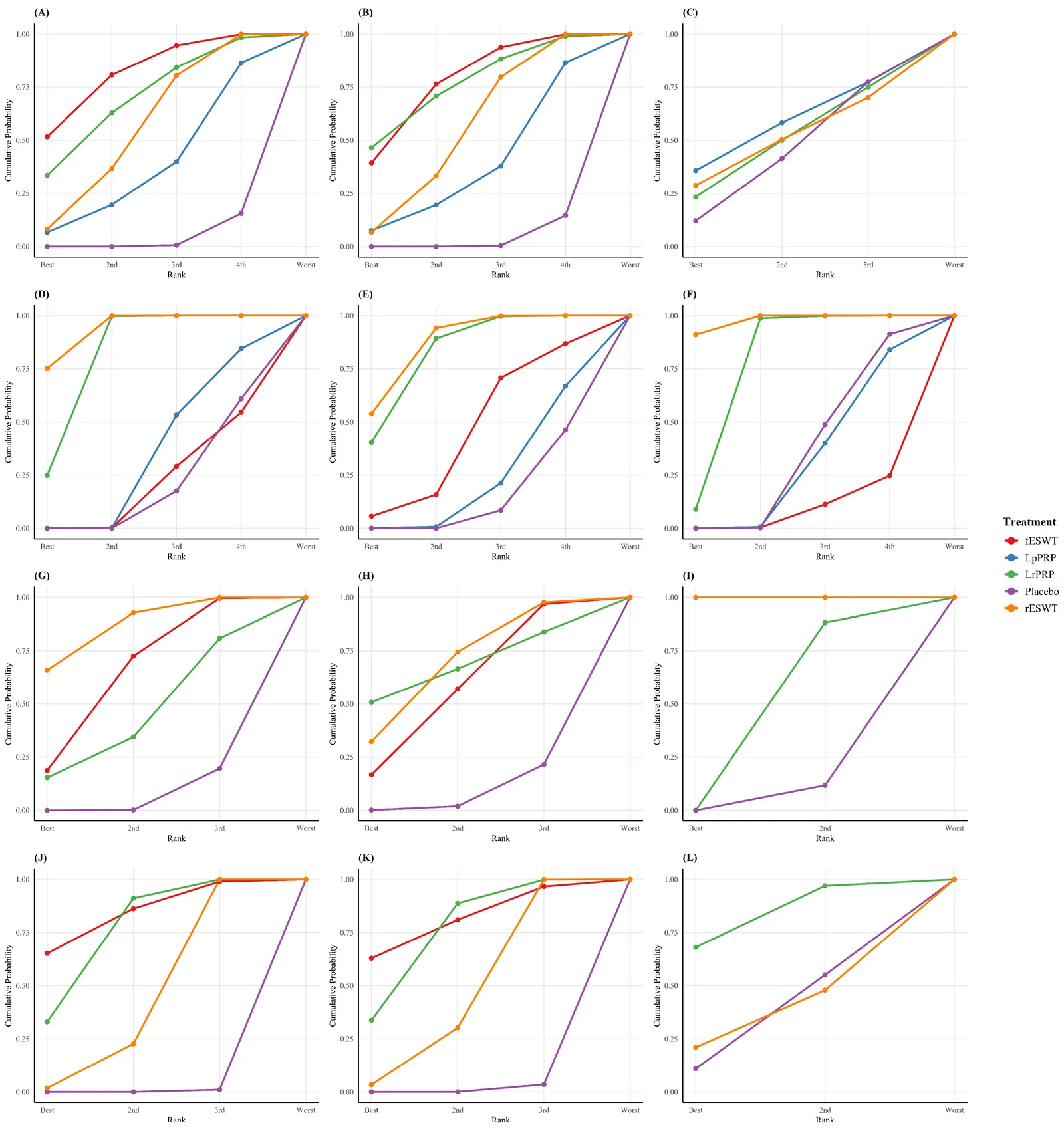
Cumulative rank probability (rankogram) for each treatment. (**A**) Grip strength; (**B**) Grip strength (≤3 months); (**C**) Grip strength (≥6 months); (**D**) DASH; (**E**) DASH (≤3 months); (**F**) DASH (≥6 months); (**G**) VAS; (**H**) VAS (≤3 months); (**I**) VAS (≥6 months); (**J**) PRTEE; (**K**) PRTEE (≤3 months); (**L**) PRTEE (≥6 months). DASH, Disabilities of the Arm, Shoulder and Hand; PRTEE, Patient-Rated Tennis Elbow Evaluation; VAS, Visual Analogue Scale.

**Figure 4:**
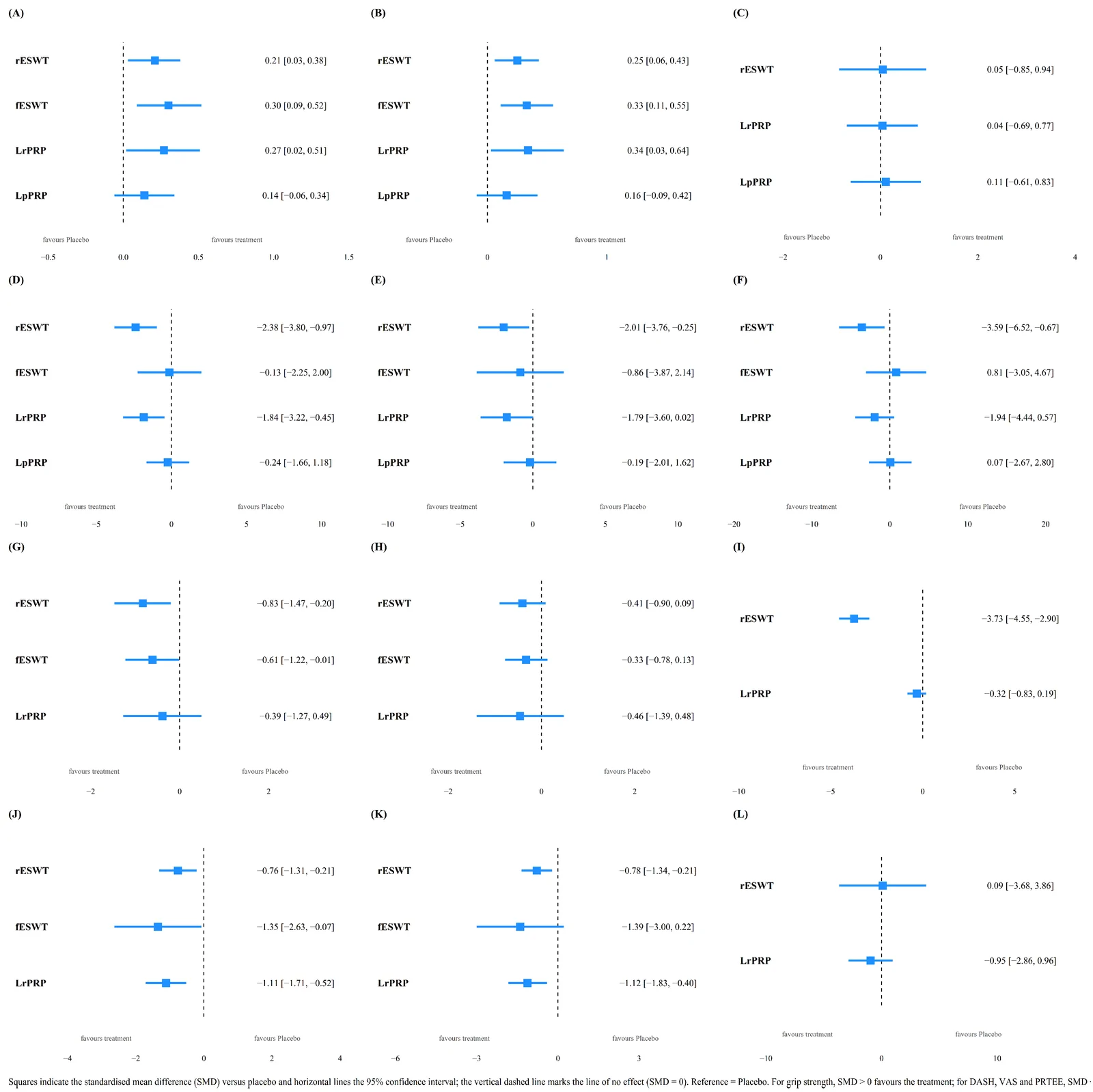
Forest plots of the standardized mean difference and 95% confidence interval for each treatment compared with placebo. (**A**) Grip strength; (**B**) Grip strength (≤3 months); (**C**) Grip strength (≥6 months); (**D**) DASH; (**E**) DASH (≤3 months); (**F**) DASH (≥6 months); (**G**) VAS; (**H**) VAS (≤3 months); (**I**) VAS (≥6 months); (**J**) PRTEE; (**K**) PRTEE (≤3 months); (**L**) PRTEE (≥6 months). In each panel, the blue square indicates the standardized mean difference (SMD) versus placebo and the horizontal line the 95% confidence interval; the vertical dashed line marks the line of no effect (SMD = 0). Placebo is the reference treatment in every panel. For grip strength (**A**–**C**) an SMD > 0 favours the active treatment; for DASH, VAS and PRTEE (**D**–**L**) an SMD < 0 favours the active treatment. DASH, Disabilities of the Arm, Shoulder and Hand; PRTEE, Patient-Rated Tennis Elbow Evaluation; VAS, Visual Analogue Scale.

The comparative efficacy of various interventions for enhancing grip strength is depicted through a league table and network plot (Table 1A and Figure 2A). According to the treatment hierarchy established by SUCRA values, fESWT emerged as the most superior intervention (SUCRA: 82.6), followed by LrPRP (71.4) (Figure 3A and Table S8). When compared with placebo, statistically significant improvements were demonstrated by fESWT (SMD: 0.30; 95% CI: 0.09 to 0.52), LrPRP (SMD: 0.27; 95% CI: 0.02 to 0.51), and rESWT (SMD: 0.21; 95% CI: 0.03 to 0.38) (Figure 4A).

In the subgroup analysis based on follow-up duration, for follow-up periods of 3 months or less, fESWT was ranked as the most effective intervention (SUCRA: 78.4), followed by LrPRP (SUCRA: 76.1) (Table 1B and Table S8). When compared with placebo, statistically significant improvements in grip strength were demonstrated by LrPRP (SMD: 0.34; 95% CI: 0.03 to 0.64), fESWT (SMD: 0.33; 95% CI: 0.11 to 0.55), and rESWT (SMD: 0.25; 95% CI: 0.06 to 0.43).

For follow-up periods of 6 months or more, no treatment retained a significant advantage: LpPRP ranked highest (SUCRA: 57.0), followed by rESWT (50.2) and LrPRP (49.4), but every 95% confidence interval for the comparison with placebo included zero, and no fESWT trial reported grip strength at this time point (Table 1C and Table S8).

### 3.5. Effects on Upper-Limb Function Measured by DASH

The network configuration and the associated league table detailing the comparative impact of interventions on upper-limb function (DASH) are illustrated in Table 1D and Figure 2D. According to the treatment hierarchy established by SUCRA values, rESWT was identified as the most superior intervention (SUCRA: 92.9), followed by LrPRP (77.4) (Figure 3D and Table S8). In comparisons with the placebo group, rESWT demonstrated a statistically significant reduction in DASH scores (SMD: −2.38; 95% CI: −3.80 to −0.97). Similarly, LrPRP (SMD: −1.84; 95% CI: −3.22 to −0.45) was significantly more effective than placebo. However, neither LpPRP (SMD: −0.24; 95% CI: −1.66 to 1.18) nor fESWT (SMD: −0.13; 95% CI: −2.25 to 2.00) demonstrated a statistically significant advantage over placebo (Figure 4D).

In the subgroup analysis based on follow-up duration, for follow-up periods of 3 months or less, rESWT was ranked as the most effective intervention (SUCRA: 82.2), followed by LrPRP (SUCRA: 74.6). When compared with placebo, a statistically significant improvement in DASH scores was demonstrated by rESWT (SMD: −2.01; 95% CI: −3.76 to −0.25) (Table 1E and Table S8).

For follow-up periods of 6 months or more, the rankings identified rESWT as the most effective intervention (SUCRA: 94.0), followed by LrPRP (SUCRA: 70.7). rESWT maintained a statistically significant superiority over placebo (SMD: −3.59; 95% CI: −6.52 to −0.67), whereas the difference between LrPRP and placebo was no longer significant (SMD: −1.94; 95% CI: −4.44 to 0.57). Conversely, fESWT was ranked as the least effective intervention at this stage (SUCRA: 19.9). Other modalities did not exhibit statistically significant differences during this late phase of follow-up (Table 1F and Table S8).

### 3.6. Effects on Pain at Rest Measured by VAS

The network configuration and the associated league table for pain at rest (VAS) are illustrated in Table 1G and Figure 2G. Because no eligible trial assessing VAS employed leukocyte-poor PRP, this network comprised four treatment nodes (fESWT, rESWT, LrPRP and placebo). According to the treatment hierarchy established by SUCRA values, rESWT was identified as the most superior intervention (SUCRA: 86.6), followed by fESWT (60.8) (Figure 3G and Table S8). In comparisons with the placebo group, rESWT (SMD: −0.83; 95% CI: −1.47 to −0.20) and fESWT (SMD: −0.61; 95% CI: −1.22 to −0.01) were significantly more effective, whereas LrPRP did not reach significance (SMD: −0.39; 95% CI: −1.27 to 0.49) (Figure 4G).

In the subgroup analysis based on follow-up duration, for follow-up periods of 3 months or less, rESWT remained the highest-ranked intervention (SUCRA: 69.2), although LrPRP (65.5) and fESWT (55.2) exchanged positions relative to the overall analysis, and no comparison with placebo reached statistical significance (Table 1H and Table S8).

For follow-up periods of 6 months or more, only three trials contributed and the network contained no closed loop, so inconsistency could not be evaluated. rESWT was ranked highest (SUCRA: 100.0) and was significantly superior to placebo (SMD: −3.73; 95% CI: −4.55 to −2.90), whereas LrPRP did not differ significantly from placebo (SMD: −0.32; 95% CI: −0.83 to 0.19) (Table 1I and Table S8); these estimates rest on very limited evidence and should be interpreted with caution. No trial contributing to the VAS network was judged to be at high risk of bias, so the sensitivity analysis left these estimates unchanged (Table S5).

### 3.7. Effects on Disease-Specific Function Measured by PRTEE

The network configuration and the associated league table for disease-specific function (PRTEE) are illustrated in Table 1J and Figure 2J. As for VAS, no eligible trial assessing PRTEE employed leukocyte-poor PRP, so this network also comprised four treatment nodes. According to the treatment hierarchy established by SUCRA values, fESWT was identified as the most superior intervention (SUCRA: 81.9), followed by LrPRP (74.2) (Figure 3J and Table S8). All three active treatments were significantly superior to placebo: fESWT (SMD: −1.35; 95% CI: −2.63 to −0.07), LrPRP (SMD: −1.11; 95% CI: −1.71 to −0.52) and rESWT (SMD: −0.76; 95% CI: −1.31 to −0.21) (Figure 4J).

In the subgroup analysis based on follow-up duration, for follow-up periods of 3 months or less, fESWT again ranked highest (SUCRA: 78.9), followed by LrPRP (73.5). LrPRP (SMD: −1.12; 95% CI: −1.83 to −0.40) and rESWT (SMD: −0.78; 95% CI: −1.34 to −0.21) remained significantly superior to placebo, whereas fESWT no longer reached significance (SMD: −1.39; 95% CI: −3.00 to 0.22) (Table 1K and Table S8).

For follow-up periods of 6 months or more, the network again contained no closed loop and no treatment differed significantly from placebo, although LrPRP ranked highest (SUCRA: 78.6) (Table 1L and Table S8). In the sensitivity analysis excluding trials at high risk of bias, the estimates for focused and radial ESWT were strengthened (SMD: −1.56 and −0.97, respectively), whereas the advantage of LrPRP was abolished (SMD: 0.00; 95% CI: −0.47 to 0.46) (Table S5).

### 3.8. Safety and Adverse Events

Throughout the studies analyzed, no serious adverse events were reported, and the evaluated therapies including rESWT, fESWT, LrPRP, and LpPRP were generally well tolerated. While some trials noted a temporary increase in pain following the procedure [32], no significant complications, such as skin irritation, infections, or tissue damage, were documented. Adverse events were not reported at all in 8 of the 23 trials. Because safety data were therefore not reported consistently across all included trials, these results should be interpreted with caution. Future research should prioritize systematic and standardized reporting of adverse events to better establish the long-term safety profile of these interventions.

### 3.9. Grading of Evidence

We assessed the quality of evidence for treatment efficacy, with comprehensive evaluations provided in Tables S9 and S10A–L. Confidence in the estimated effects varied markedly across comparisons: of the 86 comparisons assessed, 78 were rated low or very low, six moderate and two high, chiefly because of heterogeneity, imprecision and incoherence.

## 4. Discussion

This NMA evaluated the comparative effectiveness of various interventions for LET, with a specific focus on distinguishing subtypes of ESWT (fESWT vs. rESWT) and PRP (LrPRP vs. LpPRP). Our findings indicate that rESWT demonstrated the highest efficacy for long-term upper-limb function measured by the DASH score (SUCRA: 92.9 overall; 94.0 in the long term) and for pain at rest (SUCRA: 86.6). Conversely, fESWT was the most effective intervention for short-term grip strength (SUCRA: 78.4) and for disease-specific function measured by PRTEE (SUCRA: 81.9), whereas LrPRP ranked as a secondary option whose advantage was not robust to the exclusion of trials at high risk of bias. These results suggest that the physical and biological characteristics of these specific subtypes significantly influence clinical outcomes, providing a more granular framework for individualized treatment strategies [8,11].

The findings of this study align with and expand upon the existing literature regarding the general efficacy of ESWT and PRP [43,44,45]. However, recent NMAs have often failed to find clear evidence of superiority over placebo, likely due to a lack of granularity in treatment classification [46]. A 2026 network meta-analysis by Jin et al. stratified ESWT by device type and energy level and likewise found that outcomes vary across ESWT subtypes, although its principal conclusion emphasised energy level, with low-energy protocols appearing most favourable [47]. Some rankings differ from ours—Jin et al. identified focused ESWT as optimal for long-term pain and radial ESWT for PRTEE, whereas we observed the opposite pattern—which may reflect their inclusion of cohort studies, the pooling of most outcomes at a single post-treatment time point, and the difficulty of converting radial pressure values into energy flux density. Our analysis differs in that it is restricted to randomised trials, resolves both ESWT and PRP into their physical and biological subtypes within a single network, and stratifies every outcome by follow-up window; conversely, we did not stratify by energy level, which remains an important direction for future work. Specifically, our results demonstrate that ESWT subtypes show temporal and outcome-specific divergence in efficacy, with rESWT favoured for pain and long-term upper-limb function and fESWT favoured for short-term grip strength and disease-specific function. This underscores that prioritizing disease-specific outcome measures and specific formulations is essential for identifying true clinical efficacy in lateral elbow tendinopathy.

The distinction between rESWT and fESWT provides critical clinical insight. In this study, rESWT emerged as the most effective modality for long-term upper-limb function (DASH) and for pain at rest, likely due to its characteristic energy distribution across a larger superficial area. This pattern is ideally suited for the anatomy of the common extensor origin, where it induces the selective destruction of acetylcholine receptors (AChRs) at the neuromuscular junction without causing permanent injury to muscle fibers or pre-synaptic structures. In one study, the compound muscle action potential (CMAP) amplitude was observed to decrease immediately after treatment, only to gradually normalize within 8 weeks through a reinnervation process [48]. This “biological reset”—characterized by temporary degeneration followed by structural remodeling of the entire superficial muscle unit—serves as the key driver for the sustained improvements in complex daily tasks captured by the DASH score. In addition, the wider superficial field of radial devices [39] may over-stimulate peripheral nociceptors and thereby blunt nociceptive transmission, a mechanism proposed to underlie the rapid analgesia observed with radial devices [49].

In contrast, fESWT delivers high instantaneous pressure and potent stress gradients to a deep focal zone, activating mechanotransduction pathways and upregulating growth factors such as bone morphogenetic protein-2 (BMP-2) and vascular endothelial growth factor (VEGF) [50]. Because this response is concentrated at the enthesis itself rather than distributed superficially, it may act preferentially on the diseased tissue, which is consistent with the advantage of fESWT for short-term grip strength and for the disease-specific PRTEE score. Whether this focal action accounts for the temporal and outcome-specific divergence we observed between the two devices remains to be established.

Regarding PRP, our classification into leukocyte-rich and leukocyte-poor versions revealed that LrPRP was more consistently associated with functional improvement than LpPRP. This pattern is biologically plausible, being potentially driven by the complex cellular interplay between platelets and leukocytes, which may orchestrate a more robust healing cascade than platelets alone. The release of pro-inflammatory cytokines, such as IL-1β and TNF-α, by the neutrophils and monocytes present in LrPRP serves to re-initiate the inflammatory phase within the stagnant biological environment of chronic tendinopathy, while the presence of monocytes facilitates the critical M1-to-M2 macrophage polarization required for the transition from initial inflammation to effective tissue remodeling [51]. In contrast, LpPRP may lack these essential cellular mediators, potentially leading to a weaker or less sustained regenerative signal in the chronic degenerative environment of the extensor tendons. This interpretation must, however, be advanced with caution: only three LrPRP trials contributed to the networks, the long-term difference between LrPRP and placebo did not reach significance, and the LrPRP advantage disappeared when trials at high risk of bias were excluded (Table S5). The mechanistic account above should therefore be regarded as a hypothesis to be tested in adequately powered head-to-head trials rather than as an established explanation.

PRTEE was specifically developed and validated for lateral epicondylitis, whereas DASH captures broader upper-limb function and may be diluted by unaffected activities or by symptoms elsewhere in the limb; disease-specific and generic instruments therefore need not respond identically to the same intervention. The concordant superiority of ESWT across DASH, PRTEE and VAS nevertheless strengthens confidence that the observed benefits are genuine rather than instrument-dependent. From an implementation perspective, radial ESWT devices are comparatively inexpensive and widely available, and non-invasive ESWT avoids the injection-related consumable costs, procedural time and infection risk of PRP; in settings where resources or access to blood-processing facilities are limited, these considerations further favour ESWT as a first-line option. This NMA has several strengths, most notably the granular classification of ESWT and PRP, which avoids the “lumping bias” seen in previous evidence syntheses. We employed a rigorous methodology with a comprehensive search and ranking by SUCRA to provide a clear hierarchy of treatment efficacy.

### Study Limitations

Several limitations must be acknowledged. The global analysis identified significant inconsistency for grip strength and for pain at rest, suggesting that these specific rankings should be interpreted with caution. This inconsistency may stem from the inherent variability of grip strength as a metric; it is highly susceptible to factors such as pain-induced inhibition, specific joint positioning during measurement, and the patient’s subjective effort, all of which can introduce noise into the data. Egger’s test was also significant for the VAS networks, so small-study effects cannot be excluded for the pain outcome. Additionally, the heterogeneity in symptom duration and treatment protocols across the included studies may have influenced the stability of the network. Furthermore, while the CINeMA assessment revealed ‘low’ or ‘very low’ confidence levels for most comparisons, this reflects an inherent limitation of NMA arising from a relative scarcity of direct head-to-head RCTs between specific subtypes. This underscores the critical need for future primary research directly comparing focused vs. radial ESWT and leukocyte-rich vs. leukocyte-poor PRP. In a sensitivity analysis excluding the three trials at high risk of bias, the direction, significance and ranking of both focused and radial ESWT were fully preserved, whereas the advantage of leukocyte-rich PRP was attenuated and lost significance for grip strength, DASH and PRTEE (Table S5); the leukocyte-rich PRP evidence base therefore rests largely on those three trials and should be interpreted as preliminary. The long-term networks for VAS and PRTEE contained no closed loop and only two to four contributing trials, so those estimates are correspondingly fragile. Safety data were also reported inconsistently across trials, precluding any quantitative synthesis of harms. Finally, long-term significance over placebo was not achieved for grip strength at 6 months or beyond, likely due to the favorable natural history of tennis elbow over time.

## 5. Conclusions

This study provides a refined hierarchy of treatment efficacy for lateral elbow tendinopathy, highlighting that clinical outcomes are significantly influenced by the specific subtypes of ESWT and PRP. Radial ESWT was the most effective modality for pain at rest and for long-term upper-limb function, whereas focused ESWT was superior for short-term grip strength and for disease-specific function measured by PRTEE. Leukocyte-rich PRP ranked as a secondary option, but its advantage was not robust to the exclusion of trials at high risk of bias and requires confirmation. These findings underscore the importance of granular treatment classification, suggesting that clinical selection should be strategically tailored to a patient’s specific recovery priorities—balancing immediate symptomatic relief with sustained functional return. Future research should prioritize adequately powered head-to-head trials of ESWT and PRP subtypes using disease-specific outcome measures.

## Figures and Tables

**Table 1:** League tables of network meta-analysis estimates (standardized mean differences with 95% confidence intervals) for all outcomes and follow-up windows. Each cell shows the SMD (95% CI) of the column-defining treatment relative to the row-defining treatment. For grip strength (**A**–**C**), SMD > 0 favours the column-defining treatment. For DASH (**D**–**F**), VAS (**G**–**I**) and PRTEE (**J**–**L**), SMD < 0 favours the column-defining treatment. VAS denotes pain at rest. Because no eligible trial assessing VAS or PRTEE employed leukocyte-poor PRP, panels (**G**–**L**) exclude that node; in addition, no focused ESWT trial reported either outcome at 6 months or beyond, so panels (**I**,**L**) contain only three nodes and rest on limited long-term evidence, which should be interpreted with caution. Bold values indicate statistically significant comparisons (95% CI does not cross 0). Shaded cells on the diagonal identify the treatment defining the corresponding row and column.

**(A) Grip Strength**								
LpPRP	0.07 (−0.20, 0.33)			0.16 (−0.13, 0.45)		−0.14 (−0.34, 0.06)		0.13 (−0.19, 0.44)
−0.07 (−0.33, 0.20)	rESWT			0.09 (−0.09, 0.27)		**−0.21 (−0.38, −0.03)**		0.06 (−0.17, 0.29)
−0.16 (−0.45, 0.13)	−0.09 (−0.27, 0.09)			fESWT		**−0.30 (−0.52, −0.09)**		−0.04 (−0.32, 0.25)
0.14 (−0.06, 0.34)	**0.21 (0.03, 0.38)**			**0.30 (0.09, 0.52)**		Placebo		**0.27 (0.02, 0.51)**
−0.13 (−0.44, 0.19)	−0.06 (−0.29, 0.17)			0.04 (−0.25, 0.32)		**−0.27 (−0.51, −0.02)**		LrPRP
**(B) Grip strength (≤3 months)**								
LpPRP	0.08 (−0.23, 0.40)			0.17 (−0.17, 0.50)		−0.16 (−0.42, 0.09)		0.17 (−0.22, 0.57)
−0.08 (−0.40, 0.23)	rESWT			0.08 (−0.10, 0.26)		**−0.25 (−0.43, −0.06)**		0.09 (−0.19, 0.37)
−0.17 (−0.50, 0.17)	−0.08 (−0.26, 0.10)			fESWT		**−0.33 (−0.55, −0.11)**		0.01 (−0.32, 0.33)
0.16 (−0.09, 0.42)	**0.25 (0.06, 0.43)**			**0.33 (0.11, 0.55)**		Placebo		**0.34 (0.03, 0.64)**
−0.17 (−0.57, 0.22)	−0.09 (−0.37, 0.19)			−0.01 (−0.33, 0.32)		**−0.34 (−0.64, −0.03)**		LrPRP
**(C) Grip strength (≥6 months)**								
LpPRP		−0.06 (−1.21, 1.09)			−0.11 (−0.83, 0.61)		−0.07 (−1.10, 0.96)	
0.06 (−1.09, 1.21)		rESWT			−0.05 (−0.94, 0.85)		−0.01 (−0.87, 0.86)	
0.11 (−0.61, 0.83)		0.05 (−0.85, 0.94)			Placebo		0.04 (−0.69, 0.77)	
0.07 (−0.96, 1.10)		0.01 (−0.86, 0.87)			−0.04 (−0.77, 0.69)		LrPRP	
**(D) DASH**								
LpPRP	**−2.14 (−4.04, −0.24)**			0.11 (−1.68, 1.91)		0.24 (−1.18, 1.66)		−1.60 (−3.54, 0.34)
**2.14 (0.24, 4.04)**	rESWT			2.25 (−0.07, 4.58)		**2.38 (0.97, 3.80)**		0.54 (−0.97, 2.06)
−0.11 (−1.91, 1.68)	−2.25 (−4.58, 0.07)			fESWT		0.13 (−2.00, 2.25)		−1.71 (−4.16, 0.74)
−0.24 (−1.66, 1.18)	**−2.38 (−3.80, −0.97)**			−0.13 (−2.25, 2.00)		Placebo		**−1.84 (−3.22, −0.45)**
1.60 (−0.34, 3.54)	−0.54 (−2.06, 0.97)			1.71 (−0.74, 4.16)		**1.84 (0.45, 3.22)**		LrPRP
**(E) DASH (≤3 months)**								
LpPRP	−1.81 (−4.19, 0.57)			−0.67 (−3.62, 2.28)		0.19 (−1.62, 2.01)		−1.59 (−4.09, 0.90)
1.81 (−0.57, 4.19)	rESWT			1.14 (−1.81, 4.09)		**2.01 (0.25, 3.76)**		0.22 (−1.58, 2.01)
0.67 (−2.28, 3.62)	−1.14 (−4.09, 1.81)			fESWT		0.86 (−2.14, 3.87)		−0.92 (−4.18, 2.33)
−0.19 (−2.01, 1.62)	**−2.01 (−3.76, −0.25)**			−0.86 (−3.87, 2.14)		Placebo		−1.79 (−3.60, 0.02)
1.59 (−0.90, 4.09)	−0.22 (−2.01, 1.58)			0.92 (−2.33, 4.18)		1.79 (−0.02, 3.60)		LrPRP
**(F) DASH (≥6 months)**								
LpPRP	−3.66 (−7.66, 0.34)			0.74 (−1.99, 3.47)		−0.07 (−2.80, 2.67)		−2.00 (−5.71, 1.70)
3.66 (−0.34, 7.66)	rESWT			4.40 (−0.44, 9.25)		**3.59 (0.67, 6.52)**		1.66 (−1.58, 4.89)
−0.74 (−3.47, 1.99)	−4.40 (−9.25, 0.44)			fESWT		−0.81 (−4.67, 3.05)		−2.75 (−7.35, 1.86)
0.07 (−2.67, 2.80)	**−3.59 (−6.52, −0.67)**			0.81 (−3.05, 4.67)		Placebo		−1.94 (−4.44, 0.57)
2.00 (−1.70, 5.71)	−1.66 (−4.89, 1.58)			2.75 (−1.86, 7.35)		1.94 (−0.57, 4.44)		LrPRP
**(G) VAS**								
rESWT		0.22 (−0.27, 0.71)			**0.83 (0.20, 1.47)**		0.45 (−0.64, 1.53)	
−0.22 (−0.71, 0.27)		fESWT			**0.61 (0.01, 1.22)**		0.23 (−0.84, 1.30)	
**−0.83 (−1.47, −0.20)**		**−0.61 (−1.22, −0.01)**			Placebo		−0.39 (−1.27, 0.49)	
−0.45 (−1.53, 0.64)		−0.23 (−1.30, 0.84)			0.39 (−0.49, 1.27)		LrPRP	
**(H) VAS (≤3 months)**								
rESWT		0.08 (−0.28, 0.44)			0.41 (−0.09, 0.90)		−0.05 (−1.11, 1.01)	
−0.08 (−0.44, 0.28)		fESWT			0.33 (−0.13, 0.78)		−0.13 (−1.17, 0.91)	
−0.41 (−0.90, 0.09)		−0.33 (−0.78, 0.13)			Placebo		−0.46 (−1.39, 0.48)	
0.05 (−1.01, 1.11)		0.13 (−0.91, 1.17)			0.46 (−0.48, 1.39)		LrPRP	
**(I) VAS (≥6 months)**								
rESWT			**3.73 (2.90, 4.55)**			**3.41 (2.44, 4.38)**		
**−3.73 (−4.55, −2.90)**			Placebo			−0.32 (−0.83, 0.19)		
**−3.41 (−4.38, −2.44)**			0.32 (−0.19, 0.83)			LrPRP		
**(J) PRTEE**								
rESWT		−0.59 (−1.74, 0.57)			**0.76 (0.21, 1.31)**		−0.35 (−0.98, 0.28)	
0.59 (−0.57, 1.74)		fESWT			**1.35 (0.07, 2.63)**		0.24 (−1.08, 1.55)	
**−0.76 (−1.31, −0.21)**		**−1.35 (−2.63, −0.07)**			Placebo		**−1.11 (−1.71, −0.52)**	
0.35 (−0.28, 0.98)		−0.24 (−1.55, 1.08)			**1.11 (0.52, 1.71)**		LrPRP	
**(K) PRTEE (≤3 months)**								
rESWT		−0.61 (−2.12, 0.89)			**0.78 (0.21, 1.34)**		−0.34 (−1.04, 0.35)	
0.61 (−0.89, 2.12)		fESWT			1.39 (−0.22, 3.00)		0.27 (−1.39, 1.93)	
**−0.78 (−1.34, −0.21)**		−1.39 (−3.00, 0.22)			Placebo		**−1.12 (−1.83, −0.40)**	
0.34 (−0.35, 1.04)		−0.27 (−1.93, 1.39)			**1.12 (0.40, 1.83)**		LrPRP	
**(L) PRTEE (≥6 months)**								
rESWT			−0.09 (−3.86, 3.68)			−1.04 (−4.29, 2.20)		
0.09 (−3.68, 3.86)			Placebo			−0.95 (−2.86, 0.96)		
1.04 (−2.20, 4.29)			0.95 (−0.96, 2.86)			LrPRP		

Abbreviations: CI, confidence interval; DASH, Disabilities of the Arm, Shoulder and Hand; fESWT, focused extracorporeal shockwave therapy; LpPRP, leukocyte-poor platelet-rich plasma; LrPRP, leukocyte-rich platelet-rich plasma; PRTEE, Patient-Rated Tennis Elbow Evaluation; rESWT, radial extracorporeal shockwave therapy; SMD, standardized mean difference; VAS, Visual Analogue Scale.

## Data Availability

The raw data supporting the conclusions of this article will be made available by the authors on request.

## Supplementary Materials

The following supporting information can be downloaded at: https://www.mdpi.com/article/10.3390/jcm15176679/s1, Table S1: PRISMA-NMA checklist of items to include when reporting a systematic review involving a network meta-analysis; Table S2: Search strategy; Table S3: Classification of platelet-rich plasma (PRP) formulations in the included trials; Table S4: Heterogeneity statistics for each treatment network; Table S5: Sensitivity analysis excluding trials at high risk of bias; Table S6: Main outcomes of the studies included in this systematic review and meta-analysis; Figure S1: Risk of bias for randomized controlled trials assessed using the Cochrane Risk of Bias 2.0 Tool; Figure S2: Funnel plots and Egger’s tests to detect publication bias; Table S7A: Results of local inconsistency tests (node-splitting analysis); Table S7B: Global inconsistency assessment (design-by-treatment interaction model); Table S8: SUCRA values of treatments for tennis elbow; Table S9: Indirectness for all studies; Table S10: Confidence in Network Meta-analysis (CINeMA) final report.
