# Supplementary material for: Comparative Effectiveness of Focused Versus Radial Extracorporeal Shockwave Therapy and Leukocyte-Poor Versus Leukocyte-Rich Platelet-Rich Plasma for Lateral Epicondylitis: A Network Meta-Analysis
Source: J Clin Med. 2026 Aug 28;15(17):6679. doi: 10.3390/jcm15176679 (PMC13566096; doi:10.3390/jcm15176679)
Supplement: Supplementary file 1 [file jcm-15-06679-s001.zip › jcm-4465150-supplementary.pdf]

Supplementary Material

Table S1. PRISMA-NMA Checklist of Items to Include When Reporting A Systematic Review Involving a Network Meta-analysis

Table S2. Search strategy

Table S3. Classification of platelet-rich plasma (PRP) formulations in the included trials.

Table S4. Heterogeneity statistics for each treatment network.

Table S5. Sensitivity analysis excluding trials at high risk of bias.

Table S6. Main outcomes of the studies included in this systematic review and meta-analysis

Figure S1. Risk of bias for randomized controlled trials assessed using the Cochrane Risk of Bias 2.0 Tool.

Figure S2. Funnel plots and Egger's tests to detect publication bias

Table S7A. Results of local inconsistency tests (node-splitting analysis)

Table S7B. Global inconsistency assessment (design-by-treatment interaction model).

Table S8. SUCRA values of treatments for tennis elbow.

Table S9. Indirectness for all studies

Table S10. Confidence in Network Meta-analysis (CINeMA) final report

Table S1. PRISMA-NMA Checklist of Items to Include When Reporting A Systematic Review Involving a Network Meta-analysis

Section/Topic	Item #	Checklist Item	Reported on Page # (section)
**TITLE**			
Title	1	Identify the report as a systematic review *incorporating a network meta-analysis (or related form of meta-analysis).*	**p. 1 (Title page)**
**ABSTRACT**			
Structured summary	2	Provide a structured summary including, as applicable:<br>**Background:** main objectives<br>**Methods:** data sources; study eligibility criteria, participants, and interventions; study appraisal; and *synthesis methods, such as network meta-analysis.*<br>**Results:** number of studies and participants identified; summary estimates with corresponding confidence/credible intervals; *treatment rankings may also be discussed. Authors may choose to summarize pairwise comparisons against a chosen treatment included in their analyses for brevity.*<br>**Discussion/Conclusions:** limitations; conclusions and implications of findings.<br>**Other:** primary source of funding; systematic review registration number with registry name.	**pp. 1-2 (Abstract)**
**INTRODUCTION**			
Rationale	3	Describe the rationale for the review in the context of what is already known*, including mention of why a network meta-analysis has been conducted.*_	**pp. 3-4 (Section 1)**
Objectives	4	Provide an explicit statement of questions being addressed, with reference to participants, interventions, comparisons, outcomes, and study design (PICOS).	**p. 4 (Section 1)**
**METHODS**			
Protocol and registration	5	Indicate whether a review protocol exists and if and where it can be accessed (e.g., Web address); and, if available, provide registration information, including registration number.	**pp. 4-5 (Section 2.1)**
Eligibility criteria	6	Specify study characteristics (e.g., PICOS, length of follow-up) and report characteristics (e.g., years considered, language, publication status) used as	**pp. 5-6 (Section 2.3); Table S3**

		criteria for eligibility, giving rationale. *Clearly describe eligible treatments included in the treatment network, and note whether any have been clustered or merged into the same node (with justification).*_	
Information sources	7	Describe all information sources (e.g., databases with dates of coverage, contact with study authors to identify additional studies) in the search and date last searched.	**p. 5 (Section 2.2)**
Search	8	Present full electronic search strategy for at least one database, including any limits used, such that it could be repeated.	**p. 5 (Section 2.2); Table S2**
Study selection	9	State the process for selecting studies (i.e., screening, eligibility, included in systematic review, and, if applicable, included in the meta-analysis).	**pp. 5-6 (Sections 2.3-2.4); Figure 1 (p. 9)**
Data collection process	10	Describe method of data extraction from reports (e.g., piloted forms, independently, in duplicate) and any processes for obtaining and confirming data from investigators.	**p. 6 (Section 2.4)**
Data items	11	List and define all variables for which data were sought (e.g., PICOS, funding sources) and any assumptions and simplifications made.	**p. 6 (Section 2.4); Table S6**
**Geometry of the network**	**S1**	Describe methods used to explore the geometry of the treatment network under study and potential biases related to it. This should include how the evidence base has been graphically summarized for presentation, and what characteristics were compiled and used to describe the evidence base to readers.	**p. 7 (Section 2.5); Figure 2 (pp. 14-15)**
Risk of bias within individual studies	12	Describe methods used for assessing risk of bias of individual studies (including specification of whether this was done at the study or outcome level), and how this information is to be used in any data synthesis.	**p. 6 (Section 2.4); Figure S1**
Summary measures	13	State the principal summary measures (e.g., risk ratio, difference in means). *Also describe the use of additional summary measures assessed, such as treatment rankings and surface under the cumulative ranking curve (SUCRA) values, as well as modified approaches used to present summary findings from meta-analyses.*	**p. 7 (Section 2.5)**
Planned methods of analysis	14	Describe the methods of handling data and combining results of studies for each network meta-analysis. This should include, but not be limited to:<br>• *Handling of multi-arm trials;*<br>• *Selection of variance structure;*<br>• *Selection of prior distributions in Bayesian analyses; and*<br>• *Assessment of model fit.*	**p. 7 (Section 2.5)**
**Assessment of Inconsistency**	**S2**	Describe the statistical methods used to evaluate the agreement of direct and indirect evidence in	**p. 7 (Section 2.5); Tables**

			the treatment network(s) studied. Describe efforts taken to address its presence when found.	**S7A and S7B**
	Risk of bias across studies	15	Specify any assessment of risk of bias that may affect the cumulative evidence (e.g., publication bias, selective reporting within studies).	**p. 7 (Section 2.5); Figure S2**
	Additional analyses	16	Describe methods of additional analyses if done, indicating which were pre-specified. This may include, but not be limited to, the following:<br>• Sensitivity or subgroup analyses;<br>• Meta-regression analyses;<br>• *Alternative formulations of the treatment network; and*<br>• *Use of alternative prior distributions for Bayesian analyses (if applicable).*	**p. 7 (Section 2.5); Tables S4, S5**
**RESULTS†**				
	Study selection	17	Give numbers of studies screened, assessed for eligibility, and included in the review, with reasons for exclusions at each stage, ideally with a flow diagram.	**pp. 7-8 (Section 3.1); Figure 1 (p. 9)**
	**Presentation of network structure**	**S3**	Provide a network graph of the included studies to enable visualization of the geometry of the treatment network.	**Figure 2 (pp. 14-15)**
	**Summary of network geometry**	**S4**	Provide a brief overview of characteristics of the treatment network. This may include commentary on the abundance of trials and randomized patients for the different interventions and pairwise comparisons in the network, gaps of evidence in the treatment network, and potential biases reflected by the network structure.	**p. 8 (Section 3.1); Figure 2 (p. 14)**
	Study characteristics	18	For each study, present characteristics for which data were extracted (e.g., study size, PICOS, follow-up period) and provide the citations.	**p. 8 (Section 3.1); Tables S3, S6**
	Risk of bias within studies	19	Present data on risk of bias of each study and, if available, any outcome level assessment.	**pp. 9-10 (Section 3.2); Figure S1**
	Results of individual studies	20	For all outcomes considered (benefits or harms), present, for each study: 1) simple summary data for each intervention group, and 2) effect estimates and confidence intervals. *Modified approaches may be needed to deal with information from larger networks.*	**Table S6**
	Synthesis of results	21	Present results of each meta-analysis done, including confidence/credible intervals. *In larger networks, authors may focus on comparisons versus a particular comparator (e.g. placebo or standard care), with full findings presented in an appendix. League tables and forest plots may be considered to summarize pairwise comparisons.* If additional summary measures were explored (such as treatment rankings), these should also be presented.	**pp. 11-21 (Sections 3.4-3.8); Table 1 (pp. 11-13); Figures 3 and 4 (pp. 16-17); Tables S4, S8**

	**Exploration for inconsistency**	**S5**	Describe results from investigations of inconsistency. This may include such information as measures of model fit to compare consistency and inconsistency models, *P* values from statistical tests, or summary of inconsistency estimates from different parts of the treatment network.	**pp. 10-11 (Section 3.3); Tables S7A and S7B**
	Risk of bias across studies	22	Present results of any assessment of risk of bias across studies for the evidence base being studied.	**p. 10 (Section 3.2); Figure S2**
	Results of additional analyses	23	Give results of additional analyses, if done (e.g., sensitivity or subgroup analyses, meta-regression analyses*, alternative network geometries studied, alternative choice of prior distributions for Bayesian analyses,* and so forth).	**pp. 11-21 (Sections 3.4-3.7); Table S5**
**DISCUSSION**				
	Summary of evidence	24	Summarize the main findings, including the strength of evidence for each main outcome; consider their relevance to key groups (e.g., healthcare providers, users, and policy-makers).	**p. 21 (Section 3.9); pp. 21-25 (Section 4); Tables S9, S10**
	Limitations	25	Discuss limitations at study and outcome level (e.g., risk of bias), and at review level (e.g., incomplete retrieval of identified research, reporting bias). *Comment on the validity of the assumptions, such as transitivity and consistency. Comment on any concerns regarding network geometry (e.g., avoidance of certain comparisons).*	**pp. 25-26 (Section 4.1)**
	Conclusions	26	Provide a general interpretation of the results in the context of other evidence, and implications for future research.	**p. 26 (Section 5)**
**FUNDING**				
	Funding	27	Describe sources of funding for the systematic review and other support (e.g., supply of data); role of funders for the systematic review. This should also include information regarding whether funding has been received from manufacturers of treatments in the network and/or whether some of the authors are content experts with professional conflicts of interest that could affect use of treatments in the network.	**p. 27 (Funding)**

PICOS = population, intervention, comparators, outcomes, study design.
* Text in italics indicateS wording specific to reporting of network meta-analyses that has been added to guidance from the PRISMA statement.
† Authors may wish to plan for use of appendices to present all relevant information in full detail for items in this section.

Table S2. Search strategy

Search date: 2025.12.08 > Updated 2026.08.12

Database	Search strategy	Results
**Pubmed**	("Tennis Elbow"[Mesh] OR "tennis elbow"[tiab] OR "lateral epicondylitis"[tiab] OR "lateral elbow tendin*"[tiab] OR "lateral humeral epicondylitis"[tiab]) AND ("Extracorporeal Shockwave Therapy"[Mesh] OR extracorporeal shock wave*[tiab] OR extracorporeal shockwave*[tiab] OR ESWT[tiab] OR ECSWT[tiab] OR ECSW[tiab] OR "shock wave therap*"[tiab] OR "focused shock wave*"[tiab] OR "focused extracorporeal shock wave*"[tiab] OR fESWT[tiab] OR "radial shock wave*"[tiab] OR "radial extracorporeal shock wave*"[tiab] OR rESWT[tiab] OR "Platelet-Rich Plasma"[Mesh] OR "platelet rich plasma"[tiab] OR "platelet-rich plasma"[tiab] OR PRP[tiab] OR "Adrenal Cortex Hormones"[Mesh] OR corticosteroid*[tiab] OR glucocorticoid*[tiab] OR steroid*[tiab] OR triamcinolone[tiab] OR methylprednisolone[tiab] OR dexamethasone[tiab] OR betamethasone[tiab]) AND (randomized controlled trial[pt] OR controlled clinical trial[pt] OR randomized[tiab] OR randomised[tiab] OR randomly[tiab] OR "randomized controlled trial"[tiab] OR "randomised controlled trial"[tiab]) NOT (animals[mh] NOT humans[mh])	**369**
**EMBASE**	('tennis elbow'/exp OR 'tennis elbow':ti,ab OR 'lateral epicondylitis':ti,ab OR 'lateral elbow tendin*':ti,ab OR 'lateral	**684**

	humeral epicondylitis':ti,ab) AND ('shock wave therapy'/exp OR 'shock wave therap*':ti,ab OR 'extracorporeal shock wave*':ti,ab OR 'extracorporeal shockwave*':ti,ab OR eswt:ti,ab OR ecswt:ti,ab OR ecsw:ti,ab OR 'focused shock wave*':ti,ab OR 'focused extracorporeal shock wave*':ti,ab OR feswt:ti,ab OR 'radial shock wave*':ti,ab OR 'radial extracorporeal shock wave*':ti,ab OR reswt:ti,ab OR 'platelet rich plasma'/exp OR 'platelet rich plasma':ti,ab OR 'platelet-rich plasma':ti,ab OR prp:ti,ab OR 'adrenal cortex hormone'/exp OR corticosteroid*:ti,ab OR glucocorticoid*:ti,ab OR steroid*:ti,ab OR triamcinolone:ti,ab OR methylprednisolone:ti,ab OR dexamethasone:ti,ab OR betamethasone:ti,ab) AND ('randomized controlled trial'/exp OR 'controlled clinical trial'/exp OR randomized:ti,ab OR randomised:ti,ab OR randomly:ti,ab OR 'randomized controlled trial':ti,ab OR 'randomised controlled trial':ti,ab) NOT ('animal'/exp NOT 'human'/exp)	
**Cochrane**	Search Name:<br>Date Run: 08/12/2025 15:46:28<br>Comment:<br><br>ID Search Hits<br>#1 MeSH descriptor: [Tennis Elbow] explode all trees 615<br>#2 MeSH descriptor: [Extracorporeal Shockwave Therapy]	**465**

	explode all trees 350<br>#3 MeSH descriptor: [Platelet-Rich Plasma] explode all trees 1329<br>#4 MeSH descriptor: [Adrenal Cortex Hormones] explode all trees 19228<br>#5 "tennis elbow":ti,ab,kw OR "lateral epicondylitis":ti,ab,kw OR (lateral NEXT elbow NEXT tendin*):ti,ab,kw OR "lateral humeral epicondylitis":ti,ab,kw 1409<br>#6 extracorporeal shock wave*:ti,ab,kw<br>OR extracorporeal shockwave*:ti,ab,kw<br>OR ESWT:ti,ab,kw<br>OR ECSWT:ti,ab,kw<br>OR ECSW:ti,ab,kw<br>OR shock wave therap*:ti,ab,kw<br>OR focused shock wave*:ti,ab,kw<br>OR focused extracorporeal shock wave*:ti,ab,kw<br>OR radial shock wave*:ti,ab,kw<br>OR radial extracorporeal shock wave*:ti,ab,kw<br>OR rESWT:ti,ab,kw<br>OR fESWT:ti,ab,kw 3842<br>#7 "platelet rich plasma":ti,ab,kw<br>OR "platelet-rich plasma":ti,ab,kw<br>OR PRP:ti,ab,kw 5354<br>#8 corticosteroid*:ti,ab,kw	

	OR glucocorticoid*:ti,ab,kw<br>OR steroid*:ti,ab,kw<br>OR triamcinolone:ti,ab,kw<br>OR methylprednisolone:ti,ab,kw<br>OR dexamethasone:ti,ab,kw<br>OR betamethasone:ti,ab,kw 88746<br>#9 random*:ti,ab,kw<br>OR placebo:ti,ab,kw<br>OR "controlled trial":ti,ab,kw<br>OR "double blind":ti,ab,kw<br>OR "single blind":ti,ab,kw 1590297<br>#10 #1 OR #5 1409<br>#11 #2 OR #3 OR #4 OR #6 OR #7 OR #8 105122<br>#12 #10 AND #11 AND #9 439	
**Scopus**	TITLE-ABS-KEY ("tennis elbow" OR "lateral epicondylitis" OR "lateral elbow tendin*" OR "lateral humeral epicondylitis") AND TITLE-ABS-KEY ("extracorporeal shock wave*" OR "extracorporeal shockwave*" OR eswt OR ecswt OR ecsw OR "shock wave therap*" OR "shock waves" OR "focused shock wave*" OR "focused extracorporeal shock wave*" OR feswt OR "radial shock wave*" OR "radial extracorporeal shock wave*" OR reswt OR "platelet rich plasma" OR "platelet-rich plasma" OR prp OR corticosteroid* OR glucocorticoid* OR steroid* OR triamcinolone OR methylprednisolone OR dexamethasone OR betamethasone)	**370**

	AND TITLE-ABS-KEY (random* OR "controlled trial" OR placebo OR "double blind" OR "single blind") AND (LIMIT-TO (DOCTYPE , "ar"))	
**Web of Science**	TS=("tennis elbow" OR "lateral epicondylitis" OR "lateral elbow tendin*" OR "lateral humeral epicondylitis") AND TS=("extracorporeal shock wave*" OR "extracorporeal shockwave*" OR ESWT OR ECSWT OR ECSW OR "shock wave therap*" OR "shock waves" OR "focused shock wave*" OR "focused extracorporeal shock wave*" OR fESWT OR "radial shock wave*" OR "radial extracorporeal shock wave*" OR rESWT OR "platelet rich plasma" OR "platelet-rich plasma" OR PRP OR corticosteroid* OR glucocorticoid* OR steroid* OR triamcinolone OR methylprednisolone OR dexamethasone OR betamethasone) AND TS=(random* OR "controlled trial" OR placebo OR "double blind" OR "single blind")	**835**

Note. For CENTRAL, the interface reported 439 hits at the initial search (8 December 2025) and 472 at the update (12 August 2026); 465 unique records were exported and entered screening. The number shown in the Results column, and in Figure 1, is the number of records exported for screening.

Table S3. Classification of platelet-rich plasma (PRP) formulations in the included trials.

Study	Class.	Preparation system	Protocol (blood; spin)	Leukocyte reported	Basis of classification
Dejnek et al. (2025)	LrPRP	Mini GPS III (Biomet Inc., Warsaw, IN, USA)	27 mL; ACD-A; 3200 rpm (1740×g), 15 min	Yes	Original authors' terminology ("LR-PRP")
Şahbaz et al. (2021)	LrPRP	Mini GPS III (Biomet Inc., Warsaw, IN, USA)	27 mL; ACD-A; 3200 rpm (1740×g), 15 min	Yes	Original authors' terminology ("LR-PRP")
Kizilkurt et al. (2025)	LrPRP	CellPhi PRP CP20 (PALMED)	16.2 mL; sodium citrate; 1890×g, 10 min	Yes	Original authors' terminology ("leukocyte-rich PRP")
Lhee et al. (2025)	LpPRP	Harvest SmartPReP 2 (Harvest Technologies)	30 mL; 1500 rpm/15 min then 3000 rpm/3 min	Yes	Original authors' terminology ("leukocyte-reduced")
Linnanmäki et al. (2020)	LpPRP	Arthrex ACP Double Syringe Kit	15 mL; no anticoagulant; 1500 rpm, 5 min	System	ACP is a leukocyte-poor system
Schöffl et al. (2017)	LpPRP	Autologous conditioned plasma (ACP; Arthrex)	ACP double-syringe protocol	Yes	Original authors' terminology ("leucocyte-poor")
Montalvan et al. (2016)	LpPRP	Autologous conditioned plasma (ACP; Arthrex)	ACP double-syringe protocol	Yes	Original authors' terminology ("autologous conditioned plasma"; ACP is a leukocyte-poor system)

Classification rule. PRP arms were classified as leukocyte-rich (LrPRP) or leukocyte-poor (LpPRP) according to the original authors' explicit terminology; where terminology was not stated, classification was based on the preparation system. Six trials used the authors' explicit terminology; one (Linnanmäki 2020) was classified from the preparation system (Arthrex ACP), as pre-specified. Two reviewers classified each study independently, with complete agreement (Cohen's κ = 1.00). A numerical leukocyte concentration threshold was not applied because absolute leukocyte counts were not reported in a comparable form across the included trials.

Abbreviations: ACD-A, anticoagulant citrate dextrose solution A; ACP, autologous conditioned plasma; LpPRP, leukocyte-poor platelet-rich plasma; LrPRP, leukocyte-rich platelet-rich plasma; PRP, platelet-rich plasma.

Table S4. Heterogeneity statistics for each treatment network.

Outcome	τ^2	I^2 (%)	Q	df	P
Grip strength	0.000	6.6	32.13	30	0.362
Grip strength (≤3 months)	0.000	0.0	21.62	24	0.602
Grip strength (≥6 months)	0.218	66.8	9.02	3	0.029
DASH	3.972	91.2	260.91	23	<0.001
DASH (≤3 months)	3.738	90.6	116.56	11	<0.001
DASH (≥6 months)	5.771	93.5	122.40	8	<0.001
VAS	0.674	86.0	142.71	20	<0.001
VAS (≤3 months)	0.320	78.2	78.34	17	<0.001
VAS (≥6 months)	0.000	0.0	0.10	1	0.755
PRTEE	0.625	81.4	86.21	16	<0.001
PRTEE (≤3 months)	0.518	79.6	50.14	10	<0.001
PRTEE (≥6 months)	2.654	90.7	21.44	2	<0.001

Note. τ^2, between-study variance; I^2, proportion of total variability due to heterogeneity; Q, Cochran's Q statistic for within-network heterogeneity with its degrees of freedom (df) and P value. VAS denotes pain at rest. Because no eligible trial assessing VAS or PRTEE employed leukocyte-poor PRP, these networks comprised four treatment nodes.

Abbreviations: DASH, Disabilities of the Arm, Shoulder and Hand; PRTEE, Patient-Rated Tennis Elbow Evaluation; VAS, Visual Analogue Scale.

Table S5. Sensitivity analysis excluding trials at high risk of bias.

Outcome (trials)	Treatment	Primary analysis, SMD (95% CI)	Excluding high-RoB trials, SMD (95% CI)	P	SUCRA (%)
Grip strength (13 → 11)	fESWT	0.30 (0.09, 0.52)	0.39 (0.16, 0.62)	<0.001	90.0
	rESWT	0.21 (0.03, 0.38)	0.35 (0.16, 0.55)	<0.001	81.9
	LpPRP	0.14 (−0.06, 0.34)	0.14 (−0.06, 0.34)	0.164	50.5
	LrPRP	0.27 (0.02, 0.51)	−0.31 (−0.67, 0.05)	0.095	1.6
DASH (9 → 7)	rESWT	−2.38 (−3.80, −0.97)	−3.16 (−4.93, −1.39)	<0.001	99.1
	fESWT	−0.13 (−2.25, 2.00)	−0.35 (−2.59, 1.90)	0.762	41.9
	LpPRP	−0.24 (−1.66, 1.18)	−0.32 (−1.81, 1.17)	0.673	42.2
	LrPRP	−1.84 (−3.22, −0.45)	−0.23 (−2.31, 1.85)	0.831	38.6
VAS at rest (7 → 7)	rESWT	−0.83 (−1.47, −0.20)	−0.83 (−1.47, −0.20)	0.010	86.6
	fESWT	−0.61 (−1.22, −0.01)	−0.61 (−1.22, −0.01)	0.047	60.8
	LrPRP	−0.39 (−1.27, 0.49)	−0.39 (−1.27, 0.49)	0.388	45.2
PRTEE (7 → 5)	fESWT	−1.35 (−2.63, −0.07)	−1.56 (−2.20, −0.91)	<0.001	99.3
	rESWT	−0.76 (−1.31, −0.21)	−0.97 (−1.29, −0.65)	<0.001	67.3
	LrPRP	−1.11 (−1.71, −0.52)	0.00 (−0.47, 0.46)	0.986	16.9

Note. Three trials judged to be at high risk of bias (Kizilkurt et al. 2025; Şahbaz et al. 2021; Sarkar et al. 2013) were removed and each network was re-estimated with the same random-effects model and effect measure as the primary analysis. Values are standardised mean differences versus placebo; for grip strength a positive value favours the active treatment, whereas for DASH, VAS and PRTEE a negative value favours the active treatment. P values and SUCRA refer to the sensitivity analysis. The number of trials before and after exclusion is given in parentheses. No trial contributing to the VAS network was at high risk of bias, so those estimates are unchanged.

Interpretation. Estimates for focused and radial ESWT were preserved or strengthened in every network, whereas the advantage of leukocyte-rich PRP was no longer statistically significant for grip strength, DASH or PRTEE once the three high-risk trials were excluded, indicating that this finding rests largely on those trials.

Abbreviations: CI, confidence interval; DASH, Disabilities of the Arm, Shoulder and Hand; fESWT, focused extracorporeal shockwave therapy; LpPRP, leukocyte-poor platelet-rich plasma; LrPRP, leukocyte-rich platelet-rich plasma; PRTEE, Patient-Rated Tennis Elbow Evaluation; rESWT, radial extracorporeal shockwave therapy; RoB, risk of bias; SMD, standardised mean difference; SUCRA, surface under the cumulative ranking curve; VAS, Visual Analogue Scale.

Table S6. Main outcomes of the studies included in this systematic review and meta-analysis

Author (Year)	Study design	Experimental group (n)	Control group (n)	Age (Mean ± SD) (Experimental/Control groups)	Female (%)	Follow-up duration	Outcome Measure	Main Finding	Adverse effects
Akınoğlu (2025)	RCT	f-ESWT (n=29)	r-ESWT (n=27)	45.1 ± 8.1 / 44 ± 9.1	44.6	0,5 wks	1. VAS<br>2. Muscle strength<br>3. DASH<br>4. SF-36	Both improved pain and function, but f-ESWT was superior for pain reduction while r-ESWT was more effective for muscle strength and functional scores.	Not reported
Kizilkurt et al. (2025)	Single-blind RCT (3-arm)	LrPRP (n=12)	Glucocorticoid (n=12); Saline (n=12)	LrPRP : 43.4 ± 6.2 / GC: 42.2 ± 10.9 / Saline: 40.7 ± 9.4	55.6	0,3,6 months	1. VAS<br>2. DASH<br>3. PRTEE<br>4. SMI Index<br>5. US Vascularity	LrPRP showed significantly better clinical outcomes (VAS, DASH, PRTEE) than GC and saline at both 3 and 6 months, while no significant differences were observed between groups in radiologic parameters (SMI index, Doppler vascularity).	None
Lhee et al. (2025)	RCT (4-arm)	LpPRP (n=55); Prolotherapy (n=56); f-ESWT (n=62)	Physiotherapy (n=58)	LpPRP: 52.4 ± 6.7 / Prolotherapy: 51.2 ± 8.9 / ESWT: 52.6 ± 6.6 / Physiotherapy : 52.1 ± 8.5	62.8	0,3,6,18,24 months	1. DASH	LpPRP and prolotherapy showed superior functional improvement (DASH) over ESWT and physiotherapy at 2-year follow-up.	Not reported
Dejnek et al. (2025)	Single-blind RCT (4-arm)	LrPRP (n=30); Corticosteroid (n=10); Hyaluronic Acid (n=10)	Saline (n=10)	LrPRP: 49 ± 6.1 / CS: 47.6 ± 8.8 / HA: 48.6 ± 6.9 / Saline: 44.9 ± 9.8 /	48.3	0,4,12,24,52 wks	1. VAS<br>2. PRTEE<br>3. DASH<br>4. Grip strength<br>5. Wrist extensor power	All injection types (LrPRP, CS, HA) provided long-term clinical improvement, but none were significantly superior to placebo (saline) at 1 year.	Not reported
Murat et al. (2024)	Double-blind RCT	r-ESWT (n=21)	Sham-ESWT (n=21)	41.7 ± 7.6 / 41.1 ± 9.3	64.3	0,2,6 wks	1. VAS<br>2. PRTEE<br>3. Grip strength<br>4. PPT<br>5. CET	rESWT showed significant superiority over Sham-ESWT in reducing pain (VAS), improving function (PRTEE), and reducing CET thickness at 1-month follow-up.	None
Kaplan et al. (2023)	Double - blind RCT (3-arm)	f-ESWT (n=30); r-ESWT (n=29)	Sham-ESWT (n=28)	f-ESWT: 49.3 ± 14.0 / r-ESWT: 44.2 ± 13.3 / Sham-ESWT: 46.0 ± 16.9	52.9	0,5,13 wks	1. PRTEE<br>2. PRTEE pain<br>3. PRTEE function	Both fESWT and rESWT are effective, but Focused ESWT is significantly superior to Radial ESWT in terms of pain relief and functional improvement.	Not reported

Aldajah et al. (2022)	RCT	r-ESWT (n=20)	Physiotherapy (n=20)	42.0 ± 7.3 / 42.4 ± 6.7	57.5	0,4 wks	1. VAS<br>2. DASH<br>3. Grip strength	rESWT is significantly more effective than conventional physiotherapy in reducing pain and functional disability while concurrently improving maximal grip strength in individuals with lateral epicondylitis.	Not reported
Stania et al. (2021)	RCT	r-ESWT (n=14)	f-ESWT (n=13)	44.3 ± 7.9 / 47.2 ± 8.7	50	1,6,12 wks	1. VAS<br>2. Grip strength<br>3. Wrist extensor strength<br>4. Wrist flexor strength	Both FSWT and RSWT are equally effective in reducing pain and improving strength for both acute and chronic LE.	None
		r-ESWT (n=15)	f-ESWT (n=16)	45.73 ± 7.89 / 49.25 ± 6.99					
Sahbaz et al. (2021)	RCT	LrPRP+PT (n=25);<br>r-ESWT+PT (n=25)	Physiotherapy (n=24)	ESWT+PT: 49.2 ± 6.4 / LrPRP +PT: 46.3 ± 6.8 / Physiotherapy : 48.0 ± 9.1	81.1	0,1,2,3,6 months	1. VAS<br>2. DASH<br>3. PRTEE<br>4. Grip strength<br>5. Pinch strength<br>6. CET thickness	LrPRP combined with stretching and eccentric strengthening exercises is superior to exercise alone or ESWT combined with exercise in providing significant long-term (6-month) improvements in pain, functionality, and handgrip strength in patients with chronic lateral epicondylitis.	Not reported
Linnanmaki et al. (2020)	RCT	LpPRP (n=40); Autologous blood (n=40)	Saline (n=39)	46.0 ± 5.0 / 46.0 ± 10.0	53.8	0,4,8,12,26, 52 wks	1. VAS<br>2. DASH<br>3. Grip strength	LpPRP or autologous blood injections did not reduce pain or improve function more effectively than a saline injection at 52 weeks of follow-up.	None
Senol Guler et al. (2018)	Double - blind RCT	r-ESWT (n=20)	Sham-ESWT (n=20)	46.1 ± 9.5 / 46.3 ± 8.1	70	0,6 wks	1. VAS<br>2. PRTEE<br>3. Grip strength<br>4. Pinch strength	Although ESWT treatment showed improvements in pain and function, there was no statistically significant difference between the ESWT and placebo groups at the 1-month follow-up.	None
Yang et al. (2017)	Single - blind RCT	r-ESWT (n=15)	Sham-ESWT (n=13)	50.9 ± 8.4 / 51.1 ± 9.5	57.1	0,6,12,24 wks	1. VAS<br>2. DASH<br>3. Grip strength	ESWT combined with physical therapy provides more significant and faster improvements in pain reduction, grip strength, and upper extremity function compared to physical therapy alone in patients with lateral epicondylosis.	None
Schöffl et al. (2017)	Double - blind RCT	LpPRP (n=18)	Saline (n=18)	52.6 ± 11.5 / 52.6 ± 11.6	50	0,4 wks, 6 months	1. DASH	Both LpPRP and placebo groups showed improvement in DASH scores at 6 months, but there was no statistically significant difference between the groups.	None
Capan et al. (2016)	Double - blind RCT	r-ESWT (n=23)	Sham-ESWT (n=22)	48.4 ± 9.0/ 46.2 ± 7.4	77.8	0,1,3 months	1. VAS<br>2. PRTEE<br>3. Grip strength	ESWT showed improvements in pain, function, and grip strength over 3 months, but it was not significantly more effective than placebo (sham rESWT).	None
Montalvan et al. (2016)	Double - blind RCT	LpPRP (n=25)	Saline (n=25)	47.0 ± 9.2 / 46.4 ± 8.6	41	0,1,3,6,12 months	1. VAS<br>2. Roles-Maudsley score	Two ultrasound-guided LpPRP injections for recent epicondylitis were not more efficacious than saline injections at 6 and 12 months. Both groups showed significant spontaneous improvement over time.	Not reported
Król et al. (2015)	RCT	f-ESWT (n=25)	r-ESWT (n=25)	47.6 ± 7.6 / 45.8 ± 7.5	52	0,1,3,6,12 wks	1. VAS<br>2. Grip strength<br>3. Wrist extensor strength<br>4. Wrist flexor strength	Successive measurements showed a gradual and significant decrease in pain (especially during activity) and a steady increase in muscle strength in both groups. No statistically significant difference was found between focused (FSWT) and radial (RSWT) shock wave therapies at the 12-week follow-up.	Not reported
Sarkar et al. (2013)	RCT	f-ESWT + PT (n = 15)	PT (n = 15)	41.9 ± 4.5 / 42.9 ± 5.3	60	0,4 wks	1. VAS<br>2. Grip strength<br>3. DASH	Low-energy FSW combined with supervised exercise provides significantly greater pain relief (69% reduction) and functional improvement compared to exercise alone at 4 weeks.	None

Collins et al. (2011)	Multicenter double - blind RCT	f-ESWT (n=82)	Sham-ESWT (n=83)	44.0 ± 7.6 / 46.0 ± 7.5	52	0,4,8 wks	1. VAS<br>2. SF-36	A single high-energy electrohydraulic ESW treatment is significantly more effective than placebo for chronic lateral epicondylitis.	None
Spacca et al. (2005)	Single-blind RCT	r-ESWT (n=31)	Sham-ESWT (n=31)	46.8 ± 9.5/ 47.0 ± 9.2	48.4	0,4 wks, 6 months	1. VAS<br>2. DASH<br>3. PFGS	Radial shock wave therapy (RSWT) is a safe and highly effective conservative treatment for chronic tennis elbow, providing significant and sustained improvements in pain relief, grip strength, and functional status over a 6-month period compared to a low-dose control group.	None
Pettrone & McCall (2005)	Multicenter double - blind RCT	f-ESWT (n=56)	Sham-ESWT (n=58)	Not reported	52.6	0,12 wks	1. VAS<br>2. Modified UEFS<br>3. Grip strength	Low-dose extracorporeal shock wave therapy without local anesthesia is a safe and effective treatment for chronic lateral epicondylitis, providing significant improvements in pain relief and functional activity compared to placebo at twelve weeks.	None
Rompe et al. (2004)	Double - blind RCT	f-ESWT (n=38)	Sham-ESWT (n=40)	45.9 ± 12.3 / 46.2 ± 11.2	48.7	0,3,12 months	1. VAS<br>2. UEFS<br>3. Grip strength<br>4. Roles-Maudsley score	Repetitive low-energy extracorporeal shock-wave treatment without local anesthesia is significantly more effective than placebo in reducing pain and improving function for chronic lateral epicondylitis in tennis players, with clinical benefits maintained for at least 12 months.	None
Chung & Wiley (2004)	Double - blind RCT	f-ESWT (n=31)	Sham-ESWT (n=29)	46.8 ± 9.2 / 45.5 ± 6.6	38.3	0,8 wks	1. VAS<br>2. PFGS	Active extracorporeal shock wave therapy (ESWT) showed no statistically significant difference in treatment success rates or improvement in pain and grip strength compared to sham therapy for previously untreated lateral epicondylitis at 8-week follow-up.	None
Speed et al. (2002)	Double - blind RCT	f-ESWT (n=40)	Sham-ESWT (n=35)	46.5 (26-70) / 48.2 (31-65)	56	0,1,2,3 months	1. VAS	Moderate-dose ESWT showed a significant placebo effect in subjects with lateral epicondylitis, but demonstrated no statistically significant clinical benefit when compared directly to sham therapy.	2 (Worsening of symptoms)

SD, Standard deviation; RCT, Randomized controlled trial; f-ESWT, Focused extracorporeal shock wave therapy; r-ESWT, Radial extracorporeal shock wave therapy; VAS, Visual analogue scale; DASH, Disabilities of the Arm, Shoulder and Hand; SF-36, Short Form-36 Health Survey; PRP, Platelet-rich plasma; GC, Glucocorticoid; PRTEE, Patient-Rated Tennis Elbow Evaluation; SMI Index, Superb Microvascular Imaging Index; US, Ultrasound; CS, Corticosteroid; HA, Hyaluronic Acid; PPT, Pressure pain threshold; CET, Common extensor tendon; PFGS, Pain-free grip strength; UEFS, Upper Extremity Functional Scale; PT, Physical therapy; LrPRP, Leukocyte-rich platelet-rich plasma; LpPRP, Leukocyte-poor platelet-rich plasma. Note. VAS analyses were restricted to VAS at rest; trials reporting pain-free grip strength rather than maximal grip strength, or lacking dispersion measures, could not be included. Outcome measures listed here are those reported by the original trials and do not necessarily indicate contribution to the corresponding network. Participant numbers are those reported for each randomised group. The total quoted in the main text (1,484) refers to participants allocated to treatments represented in the networks (focused ESWT, radial ESWT, leukocyte-rich PRP, leukocyte-poor PRP and placebo/control); the glucocorticoid, corticosteroid, hyaluronic acid, prolotherapy and autologous blood arms were not eligible treatments and are excluded from that total, as is the exercise-only arm of Şahbaz et al., which did not contribute to any comparison.

Figure S1. Risk of bias for randomized controlled trials assessed using the Cochrane Risk of Bias 2.0 Tool.

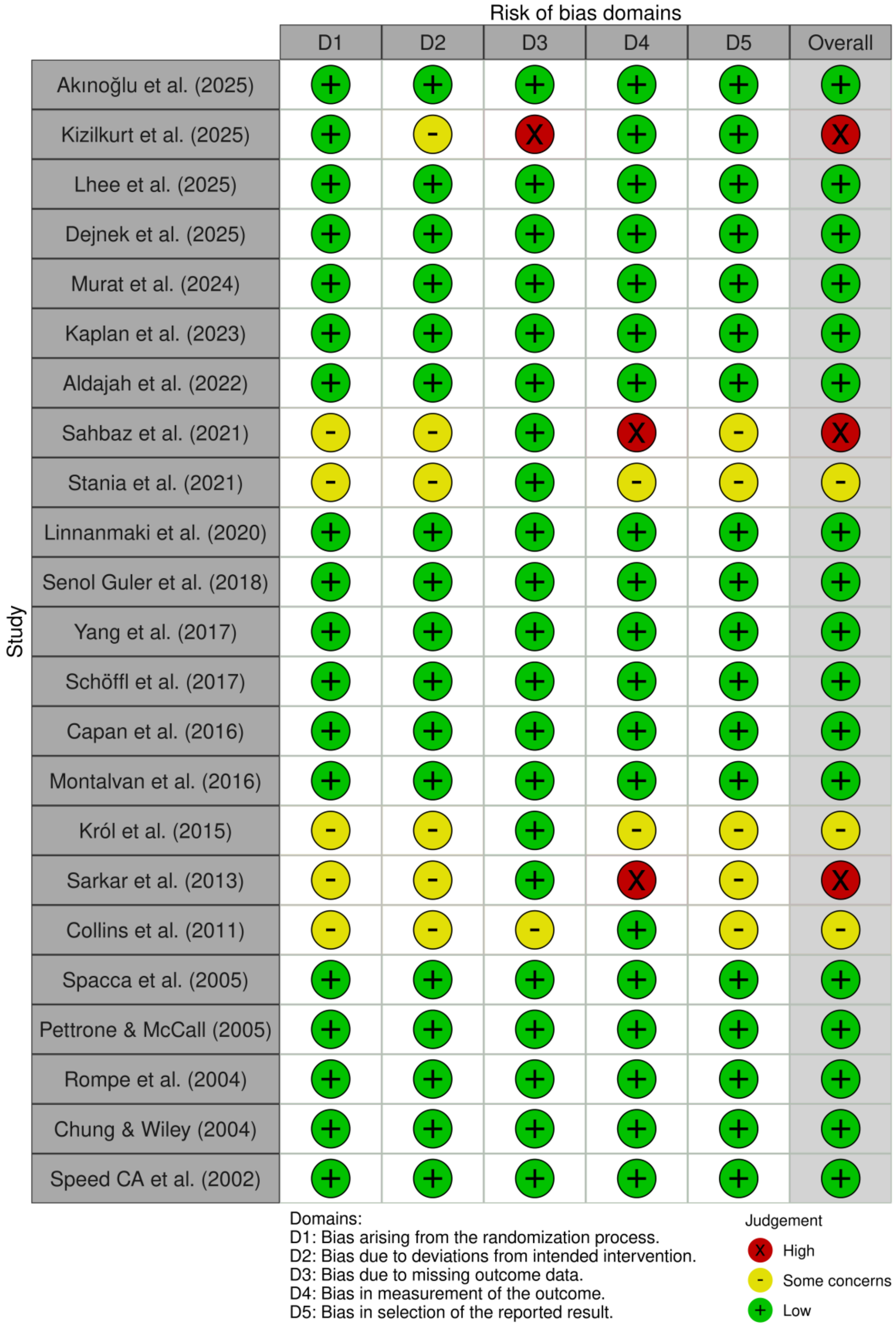

Figure S2. Funnel plots and Egger's tests to detect publication bias

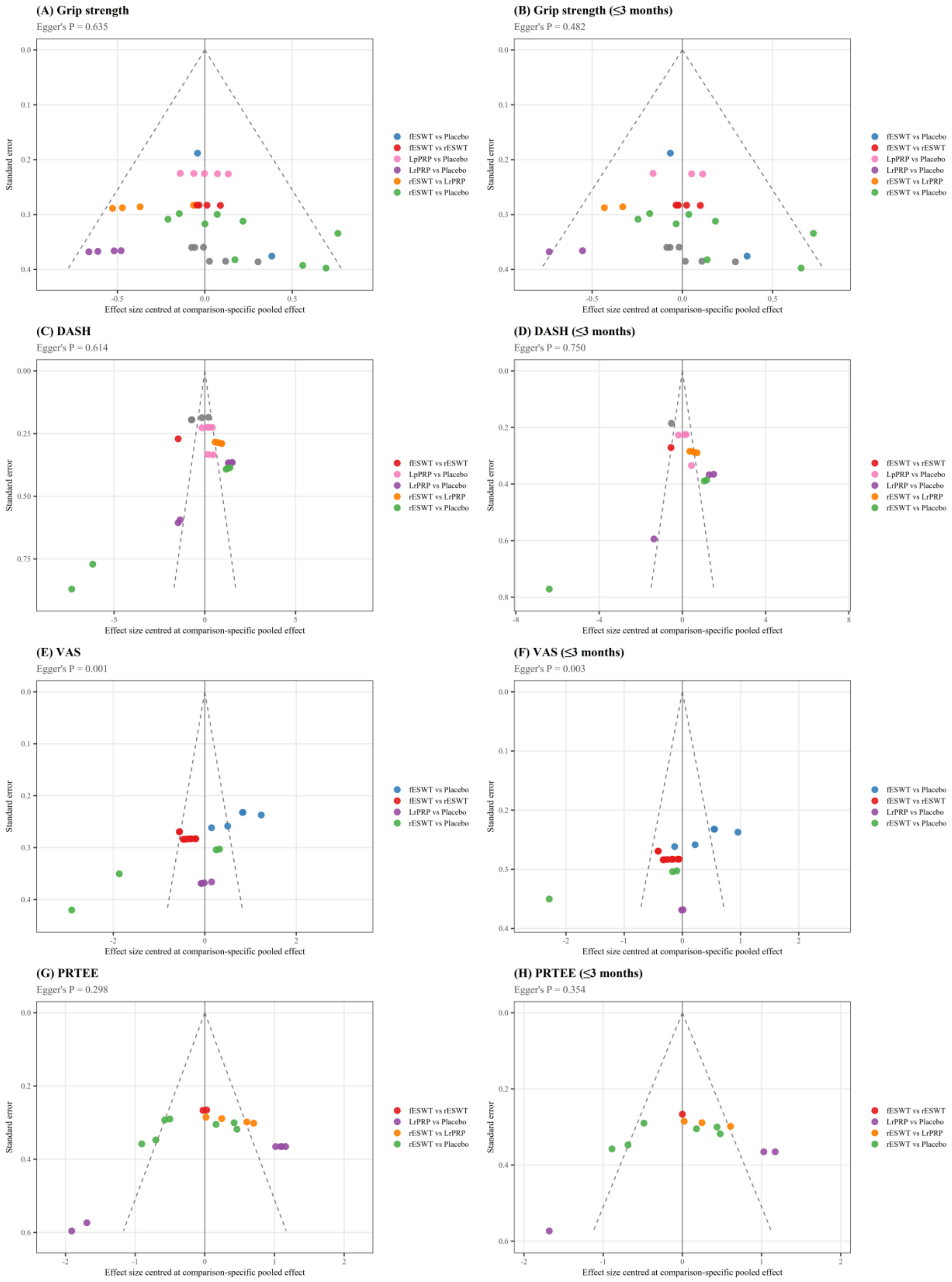

Table S7A. Results of local inconsistency tests (node-splitting analysis)

Outcome	Comparison	Direct	Indirect	Difference	P
Grip	Placebo vs fESWT	-0.357	-0.270	-0.087	0.709
	rESWT vs fESWT	-0.076	-0.163	0.087	0.709
	LrPRP vs Placebo	-0.306	0.779	-1.085	<0.001
	LrPRP vs rESWT	0.407	-0.678	1.085	<0.001
	Placebo vs rESWT	-0.399	0.150	-0.549	0.005
Grip (≤3 months)	Placebo vs fESWT	-0.348	-0.314	-0.034	0.881
	rESWT vs fESWT	-0.076	-0.110	0.034	0.881
	LrPRP vs Placebo	-0.309	0.691	-1.000	0.002
	LrPRP vs rESWT	0.350	-0.650	1.000	0.002
	Placebo vs rESWT	-0.364	0.000	-0.364	0.071
Grip (≥6 months)	LrPRP vs Placebo	-0.303	1.349	-1.652	0.053
	LrPRP vs rESWT	0.578	-1.074	1.652	0.053
	Placebo vs rESWT	-0.771	0.881	-1.652	0.053
DASH	LpPRP vs fESWT	-0.554	1.711	-2.265	0.056
	rESWT vs fESWT	-0.470	-2.735	2.265	0.056
	LpPRP vs Placebo	0.012	-2.253	2.265	0.056
	LrPRP vs Placebo	-1.040	-3.080	2.040	0.008
	LrPRP vs rESWT	-0.483	1.557	-2.040	0.008
	Placebo vs rESWT	3.088	0.399	2.689	<0.001
DASH (≤3 months)	LpPRP vs fESWT	0.004	1.353	-1.349	0.384
	rESWT vs fESWT	-0.470	-1.819	1.349	0.384
	LpPRP vs Placebo	-0.030	-1.379	1.349	0.384
	LrPRP vs Placebo	-1.034	-2.677	1.643	0.104
	LrPRP vs rESWT	-0.435	1.208	-1.643	0.104
	Placebo vs rESWT	2.556	0.576	1.980	0.038
DASH (≥6 months)	LrPRP vs Placebo	-1.076	-4.805	3.729	0.015
	LrPRP vs rESWT	-0.628	3.101	-3.729	0.015
	Placebo vs rESWT	4.176	0.447	3.729	0.015
VAS	Placebo vs fESWT	-0.111	1.949	-2.060	<0.001
	rESWT vs fESWT	0.150	-1.909	2.059	<0.001
	Placebo vs rESWT	1.798	-0.261	2.059	<0.001
VAS (≤3 months)	Placebo vs fESWT	-0.113	1.362	-1.475	0.002
	rESWT vs fESWT	0.150	-1.325	1.475	0.002
	Placebo vs rESWT	1.212	-0.263	1.475	0.002
VAS (≥6 months)	No closed loops — not estimable				
PRTEE	LrPRP vs Placebo	-0.789	-1.714	0.925	0.108

Outcome	Comparison	Direct	Indirect	Difference	P
	LrPRP vs rESWT	-0.732	0.194	-0.926	0.108
	Placebo vs rESWT	0.982	0.057	0.925	0.108
PRTEE (≤3 months)	LrPRP vs Placebo	-0.758	-1.550	0.792	0.241
	LrPRP vs rESWT	-0.629	0.163	-0.792	0.241
	Placebo vs rESWT	0.922	0.130	0.792	0.241
PRTEE (≥6 months)	No closed loops — not estimable				

Table S7B. Global inconsistency assessment (design-by-treatment interaction model).

Outcome	χ^2	df	P
Grip	19.32	2	0.0001
Grip (≤3 months)	10.08	2	0.0065
Grip (≥6 months)	9.32	1	0.0023
DASH	5.13	2	0.0769
DASH (≤3 months)	1.46	2	0.4818
DASH (≥6 months)	1.79	1	0.1815
VAS	10.22	1	0.0014
VAS (≤3 months)	8.52	1	0.0035
VAS (≥6 months)	—	0	—
PRTEE	1.93	1	0.1652
PRTEE (≤3 months)	1.10	1	0.2937
PRTEE (≥6 months)	—	0	—

Table S8. SUCRA values of treatments for tennis elbow.

	Grip strength	Grip (≤3 mo)	Grip (≥6 mo)	DASH	DASH (≤3 mo)	DASH (≥6 mo)	VAS	VAS (≤3 mo)	VAS (≥6 mo)	PRTEE	PRTEE (≤3 mo)	PRTEE (≥6 mo)
**LpPRP**	39.5	38.9	57.0	31.2	27.1	34.1	–	–	–	–	–	–
**LrPRP**	71.4	76.1	49.4	77.4	74.6	70.7	45.2	65.5	44.5	74.2	73.5	78.6
**Placebo**	2.8	3.2	43.4	20.7	18.6	31.3	7.4	10.1	5.5	0.8	1.7	34.2
**fESWT**	82.6	78.4	–	27.8	47.5	19.9	60.8	55.2	–	81.9	78.9	–
**rESWT**	53.7	53.5	50.2	92.9	82.2	94.0	86.6	69.2	100.0	43.1	45.9	37.3

Table S9. Indirectness for all studies

Author (Year)	Population	Intervention	Outcome	Comparisons	Indirectness
Akınoğlu (2025)	Low	Low	Low	Low	Low
Kizilkurt (2025)	Low	Low	Low	Low	Low
Lhee (2025)	Low	Low	Low	Low	Low
Dejnek (2025)	Low	Low	Low	Low	Low
Murat (2024)	Low	Low	Low	Low	Low
Kaplan (2023)	Low	Low	Low	Low	Low
Aldajah (2022)	Low	Low	Low	Low	Low
Stania (2021)	Low	Low	Low	Low	Low
Sahbaz (2021)	Low	Low	Low	Low	Low
Linnanmaki (2020)	Low	Low	Low	Low	Low
Senol Guler (2018)	Low	Low	Low	Low	Low
Yang (2017)	Low	Low	Low	Low	Low
Schöffl (2017)	Low	Low	Low	Low	Low
Capan (2016)	Low	Low	Low	Low	Low
Montalvan (2016)	Low	Low	Low	Low	Low
Król (2015)	Low	Low	Low	Low	Low
Sarkar (2013)	Low	Low	Low	Low	Low
Collins (2011)	Low	Low	Low	Low	Low
Spacca (2005)	Low	Low	Low	Low	Low
Pettrone (2005)	Low	Low	Low	Low	Low
Rompe (2004)	Low	Low	Low	Low	Low
Chung (2004)	Low	Low	Low	Low	Low
Speed (2002)	Low	Low	Low	Low	Low

Table S10. Confidence in Network Meta-analysis (CINeMA) final report

(A) CINeMA confidence rating for Grip strength

Comparison	Number of studies	Within-study bias	Reporting bias	Indirectness	Imprecision	Heterogeneity	Incoherence	Confidence rating
LpPRP:Placebo	5	No concerns	Low risk	No concerns	No concerns	No concerns	Major concerns	Low
LrPRP:Placebo	4	Some concerns	Low risk	No concerns	No concerns	Some concerns	Major concerns	Very low
LrPRP:rESWT	4	Some concerns	Low risk	No concerns	No concerns	No concerns	Major concerns	Very low
fESWT:Placebo	2	Some concerns	Low risk	No concerns	No concerns	No concerns	No concerns	Moderate
Placebo:rESWT	9	No concerns	Low risk	No concerns	No concerns	No concerns	Some concerns	Moderate
fESWT:rESWT	10	Some concerns	Low risk	No concerns	No concerns	No concerns	No concerns	Moderate
LpPRP:LrPRP	0	No concerns	Low risk	No concerns	No concerns	No concerns	Major concerns	Low
fESWT:LpPRP	0	No concerns	Low risk	No concerns	No concerns	Some concerns	Major concerns	Very low
LpPRP:rESWT	0	No concerns	Low risk	No concerns	No concerns	No concerns	Major concerns	Low
fESWT:LrPRP	0	Some concerns	Low risk	No concerns	No concerns	No concerns	Major concerns	Very low

(B) CINeMA confidence rating for Grip strength (<3 months)

Comparison	Number of studies	Within-study bias	Reporting bias	Indirectness	Imprecision	Heterogeneity	Incoherence	Confidence rating
LpPRP:Placebo	3	No concerns	Low risk	No concerns	No concerns	No concerns	Major concerns	Low
LrPRP:Placebo	2	Some concerns	Low risk	No concerns	No concerns	No concerns	Major concerns	Very low
LrPRP:rESWT	3	Major concerns	Low risk	No concerns	No concerns	No concerns	Major concerns	Very low
fESWT:Placebo	2	No concerns	Low risk	No concerns	No concerns	No concerns	No concerns	High
Placebo:rESWT	8	No concerns	Low risk	No concerns	No concerns	No concerns	Some concerns	Moderate
fESWT:rESWT	10	Some concerns	Low risk	No concerns	No concerns	No concerns	No concerns	Moderate
LpPRP:LrPRP	0	No concerns	Low risk	No concerns	Some concerns	No concerns	Major concerns	Very low
fESWT:LpPRP	0	No concerns	Low risk	No concerns	Some concerns	No concerns	Major concerns	Very low
LpPRP:rESWT	0	No concerns	Low risk	No concerns	No concerns	No concerns	Major concerns	Low

fESWT:LrPRP	0	Some concerns	Low risk	No concerns	No concerns	No concerns	Major concerns	Very low

(C) CINeMA confidence rating for Grip strength (>6 months)

Comparison	Number of studies	Within-study bias	Reporting bias	Indirectness	Imprecision	Heterogeneity	Incoherence	Confidence rating
LpPRP:Placebo	2	No concerns	Low risk	No concerns	Major concerns	No concerns	Major concerns	Very low
LrPRP:Placebo	2	No concerns	Low risk	No concerns	Major concerns	No concerns	Major concerns	Very low
LrPRP:rESWT	1	Some concerns	Low risk	No concerns	Major concerns	No concerns	Major concerns	Very low
Placebo:rESWT	1	No concerns	Low risk	No concerns	Major concerns	No concerns	Major concerns	Very low
LpPRP:LrPRP	0	No concerns	Low risk	No concerns	Major concerns	No concerns	Major concerns	Very low
LpPRP:rESWT	0	No concerns	Low risk	No concerns	Major concerns	No concerns	Major concerns	Very low

(D) CINeMA confidence rating for DASH

Comparison	Number of studies	Within-study bias	Reporting bias	Indirectness	Imprecision	Heterogeneity	Incoherence	Confidence rating
LpPRP:Placebo	7	No concerns	Low risk	No concerns	Some concerns	Some concerns	Some concerns	Very low
fESWT:LpPRP	4	No concerns	Low risk	No concerns	Major concerns	No concerns	Major concerns	Very low
LrPRP:Placebo	6	Some concerns	Low risk	No concerns	No concerns	Some concerns	Some concerns	Very low
LrPRP:rESWT	4	Some concerns	Low risk	No concerns	Some concerns	Some concerns	Major concerns	Very low
Placebo:rESWT	5	No concerns	Low risk	No concerns	No concerns	Some concerns	Major concerns	Very low
fESWT:rESWT	1	No concerns	Low risk	No concerns	No concerns	Some concerns	Major concerns	Very low
LpPRP:LrPRP	0	No concerns	Low risk	No concerns	No concerns	Major concerns	Major concerns	Very low
LpPRP:rESWT	0	No concerns	Low risk	No concerns	No concerns	Some concerns	Major concerns	Very low
fESWT:LrPRP	0	No concerns	Low risk	No concerns	No concerns	Major concerns	Major concerns	Very low
fESWT:Placebo	0	No concerns	Low risk	No concerns	Major concerns	No concerns	Major concerns	Very low

(E) CINeMA confidence rating for DASH (<3 months)

Comparison	Number of studies	Within-study bias	Reporting bias	Indirectness	Imprecision	Heterogeneity	Incoherence	Confidence rating
LpPRP:Placebo	4	No concerns	Low risk	No concerns	Major concerns	No concerns	No concerns	Low
fESWT:LpPRP	1	No concerns	Low risk	No concerns	Major concerns	No concerns	No concerns	Low
LrPRP:Placebo	3	Some concerns	Low risk	No concerns	No concerns	Major concerns	No concerns	Very low
LrPRP:rESWT	3	Some concerns	Low risk	No concerns	Major concerns	No concerns	No concerns	Very low
Placebo:rESWT	3	No concerns	Low risk	No concerns	No concerns	Major concerns	Major concerns	Very low
fESWT:rESWT	1	No concerns	Low risk	No concerns	Some concerns	Some concerns	No concerns	Low
LpPRP:LrPRP	0	No concerns	Low risk	No concerns	No concerns	Major concerns	No concerns	Low
LpPRP:rESWT	0	No concerns	Low risk	No concerns	No concerns	Major concerns	No concerns	Low
fESWT:LrPRP	0	Some concerns	Low risk	No concerns	Major concerns	No concerns	No concerns	Very low
fESWT:Placebo	0	No concerns	Low risk	No concerns	Major concerns	No concerns	No concerns	Low

(F) CINeMA confidence rating for DASH (>6 months)

Comparison	Number of studies	Within-study bias	Reporting bias	Indirectness	Imprecision	Heterogeneity	Incoherence	Confidence rating
LpPRP:Placebo	3	No concerns	Low risk	No concerns	Major concerns	No concerns	No concerns	Low
fESWT:LpPRP	3	No concerns	Low risk	No concerns	No concerns	Major concerns	No concerns	Low
LrPRP:Placebo	3	Some concerns	Low risk	No concerns	No concerns	Major concerns	No concerns	Very low
LrPRP:rESWT	1	Some concerns	Low risk	No concerns	Major concerns	No concerns	No concerns	Very low
Placebo:rESWT	2	No concerns	Low risk	No concerns	No concerns	Major concerns	No concerns	Low
LpPRP:LrPRP	0	No concerns	Low risk	No concerns	No concerns	Major concerns	No concerns	Low
LpPRP:rESWT	0	No concerns	Low risk	No concerns	No concerns	Major concerns	No concerns	Low
fESWT:LrPRP	0	No concerns	Low risk	No concerns	No concerns	No concerns	No concerns	High
fESWT:Placebo	0	No concerns	Low risk	No concerns	Major concerns	No concerns	No concerns	Low
fESWT:rESWT	0	No concerns	Low risk	No concerns	No concerns	Major concerns	No concerns	Low

(G)CINeMA confidence rating for VAS (rest)

Comparison	Number of studies	Within-study bias	Reporting bias	Indirectness	Imprecision	Heterogeneity	Incoherence	Confidence rating
LrPRP:Placebo	4	No concerns	Low risk	No concerns	Some concerns	Some concerns	Major concerns	Very low
Placebo:fESWT	5	No concerns	Low risk	No concerns	No concerns	Major concerns	Major concerns	Very low
Placebo:rESWT	4	No concerns	Low risk	No concerns	No concerns	Major concerns	Major concerns	Very low
fESWT:rESWT	10	Some concerns	Low risk	No concerns	Some concerns	Some concerns	Major concerns	Very low
LrPRP:fESWT	0	No concerns	Low risk	No concerns	Major concerns	No concerns	Major concerns	Very low
LrPRP:rESWT	0	No concerns	Low risk	No concerns	Major concerns	No concerns	Major concerns	Very low

(H)CINeMA confidence rating for VAS (rest) (<3 months)

Comparison	Number of studies	Within-study bias	Reporting bias	Indirectness	Imprecision	Heterogeneity	Incoherence	Confidence rating
LrPRP:Placebo	2	No concerns	Low risk	No concerns	Some concerns	Some concerns	Major concerns	Very low
Placebo:fESWT	5	No concerns	Low risk	No concerns	Some concerns	Some concerns	Major concerns	Very low
Placebo:rESWT	3	No concerns	Low risk	No concerns	Some concerns	Some concerns	Major concerns	Very low
fESWT:rESWT	10	Some concerns	Low risk	No concerns	No concerns	Major concerns	Major concerns	Very low
LrPRP:fESWT	0	No concerns	Low risk	No concerns	Major concerns	No concerns	Major concerns	Very low
LrPRP:rESWT	0	No concerns	Low risk	No concerns	Major concerns	No concerns	Major concerns	Very low

(I) CINeMA confidence rating for VAS (rest) (>6 months)

Comparison	Number of studies	Within-study bias	Reporting bias	Indirectness	Imprecision	Heterogeneity	Incoherence	Confidence rating
LrPRP:Placebo	2	No concerns	Low risk	No concerns	Some concerns	No concerns	Major concerns	Very low

Placebo:rESWT	1	No concerns	Low risk	No concerns	No concerns	No concerns	Major concerns	Low
LrPRP:rESWT	0	No concerns	Low risk	No concerns	No concerns	No concerns	Major concerns	Low

(J) CINeMA confidence rating for PRTEE

Comparison	Number of studies	Within-study bias	Reporting bias	Indirectness	Imprecision	Heterogeneity	Incoherence	Confidence rating
LrPRP:Placebo	6	No concerns	Low risk	No concerns	No concerns	Some concerns	No concerns	Moderate
LrPRP:rESWT	4	Major concerns	Low risk	No concerns	Some concerns	Some concerns	No concerns	Very low
Placebo:rESWT	7	No concerns	Low risk	No concerns	No concerns	Major concerns	No concerns	Low
fESWT:rESWT	2	No concerns	Low risk	No concerns	Some concerns	Some concerns	Major concerns	Very low
LrPRP:fESWT	0	No concerns	Low risk	No concerns	Major concerns	No concerns	Major concerns	Very low
Placebo:fESWT	0	No concerns	Low risk	No concerns	No concerns	Major concerns	Major concerns	Very low

(K) CINeMA confidence rating for PRTEE (<3 months)

Comparison	Number of studies	Within-study bias	Reporting bias	Indirectness	Imprecision	Heterogeneity	Incoherence	Confidence rating
LrPRP:Placebo	3	No concerns	Low risk	No concerns	No concerns	Major concerns	No concerns	Low
LrPRP:rESWT	3	Major concerns	Low risk	No concerns	Some concerns	Some concerns	No concerns	Very low
Placebo:rESWT	6	No concerns	Low risk	No concerns	No concerns	Major concerns	No concerns	Low
fESWT:rESWT	1	No concerns	Low risk	No concerns	Major concerns	No concerns	Major concerns	Very low
LrPRP:fESWT	0	No concerns	Low risk	No concerns	Major concerns	No concerns	Major concerns	Very low
Placebo:fESWT	0	No concerns	Low risk	No concerns	Some concerns	Some concerns	Major concerns	Very low

(L) CINeMA confidence rating for PRTEE (>6 months)

Comparison	Number of studies	Within-study bias	Reporting bias	Indirectness	Imprecision	Heterogeneity	Incoherence	Confidence rating
LrPRP:Placebo	3	No concerns	Low risk	No concerns	Major concerns	No concerns	Major concerns	Very low
LrPRP:rESWT	1	Major concerns	Low risk	No concerns	Major concerns	No concerns	Major concerns	Very low
Placebo:rESWT	0	Major concerns	Low risk	No concerns	Major concerns	No concerns	Major concerns	Very low

Note. Confidence ratings were derived using the CINeMA framework with a clinically important effect size of SMD 0.5, the 'majority' rule for within-study bias and indirectness, and a random-effects model. CINeMA implements the DerSimonian-Laird estimator of between-study variance, whereas the main analyses used REML; confidence intervals displayed within CINeMA therefore differ marginally from those in Table 1, without altering any conclusion. For the long-term VAS (rest) and PRTEE networks no closed loops of evidence were present, so incoherence could not be evaluated and was assigned 'major concerns' for all comparisons in accordance with the CINeMA framework.
